# Elucidating the Origins of High Preferential Crystal Orientation in Quasi‐2D Perovskite Solar Cells

**DOI:** 10.1002/adma.202208061

**Published:** 2022-12-05

**Authors:** Lukas E. Lehner, Stepan Demchyshyn, Kilian Frank, Alexey Minenkov, Dominik J. Kubicki, He Sun, Bekele Hailegnaw, Christoph Putz, Felix Mayr, Munise Cobet, Günter Hesser, Wolfgang Schöfberger, Niyazi Serdar Sariciftci, Markus Clark Scharber, Bert Nickel, Martin Kaltenbrunner

**Affiliations:** ^1^ Division of Soft Matter Physics Institute of Experimental Physics Johannes Kepler University Altenberger Str. 69 4040 Linz Austria; ^2^ Soft Materials Lab Linz Institute of Technology Johannes Kepler University Altenberger Str. 69 4040 Linz Austria; ^3^ Soft Condensed Matter Group Faculty of Physics Ludwig‐Maximilian University Geschwister‐Scholl‐Platz 1 80539 Munich Germany; ^4^ Center for Surface and Nanoanalytics Johannes Kepler University Altenberger Str. 69 4040 Linz Austria; ^5^ Department of Physics University of Warwick CV4 7AL Coventry UK; ^6^ Institute of Organic Chemistry Johannes Kepler University Altenberger Str. 69 4040 Linz Austria; ^7^ Linz Institute for Organic Solar Cells (LIOS) and Institute for Physical Chemistry Johannes Kepler University Altenberger Str. 69 4040 Linz Austria

**Keywords:** chloride, nucleation, perovskites, preferential orientation, 2D systems

## Abstract

Incorporating large organic cations to form 2D and mixed 2D/3D structures significantly increases the stability of perovskite solar cells. However, due to their low electron mobility, aligning the organic sheets to ensure unimpeded charge transport is critical to rival the high performances of pure 3D systems. While additives such as methylammonium chloride (MACl) can enable this preferential orientation, so far, no complete description exists explaining how they influence the nucleation process to grow highly aligned crystals. Here, by investigating the initial stages of the crystallization, as well as partially and fully formed perovskites grown using MACl, the origins underlying this favorable alignment are inferred. This mechanism is studied by employing 3‐fluorobenzylammonium in quasi‐2D perovskite solar cells. Upon assisting the crystallization with MACl, films with a degree of preferential orientation of 94%, capable of withstanding moisture levels of 97% relative humidity for 10 h without significant changes in the crystal structure are achieved. Finally, by combining macroscopic, microscopic, and spectroscopic studies, the nucleation process leading to highly oriented perovskite films is elucidated. Understanding this mechanism will aid in the rational design of future additives to achieve more defect tolerant and stable perovskite optoelectronics.

## Graphical abstract

The nucleation process to form quasi‐2D perovskites is thermodynamically controlled using the methylammonium chloride additive to achieve films with a degree of preferential orientation of 94%, capable of withstanding 97% relative humidity for 10 h without degradation. Combining microscopic, macroscopic, and spectrographic observations, the thermodynamics enabling preferential crystal growth are inferred.

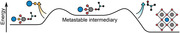

## 1 Introduction

2D and mixed‐dimensionality 2D/3D perovskites have emerged as a more stable and versatile class of materials for solar cell absorbers compared to their 3D counterparts.^[^ ^1^ ^]^ However, the insulating nature of the large organic spacer cations employed to achieve low‐dimensional structures hinders the mobility of photogenerated charges in the photoactive material. Because of this, growing films with the organic sheets aligned vertically relative to the substrate is critical to facilitate efficient charge carrier extraction.^[^ ^2^ ^]^ Preferential orientation in this class of materials has previously been induced using hot‐casting,^[^ ^3^, ^4^ ^]^ or by modifying the perovskite (PSK) precursor solution using alternate solvents, such as *N*,*N*‐dimethylacetamide (DMAc),^[^ ^2^ ^]^ or additives, such as ammonium thiocyanate (NH_4_SCN),^[^ ^5^, ^6^ ^]^ formamidinium chloride (FACl),^[^ ^7^, ^8^, ^9^ ^]^ PbCl_2_,^[^ ^10^ ^]^ and methylammonium chloride (MACl).^[^ ^11^, ^12^ ^]^

Chen et al. proposed that vertical alignment originates from the crystallization in the perovskite precursor solution starting at the liquid–air interface, rather than the rough substrate‐liquid interface or the isotropic liquid bulk.^[^ ^2^ ^]^ However, this finding was derived from perovskite films grown from a DMAc‐based solution, which favors the rapid nucleation of MAPbI_3_, thus changing the subsequent crystallization dynamics compared to more widely used solvents, such as *N*,*N*‐dimethylformamide (DMF).^[^ ^13^ ^]^ Therefore it is unclear if other methods of inducing preferential orientation are caused by the same effect.

In particular, MACl has been used to control the crystallization in many high‐performing solar cells in recent years.^[^ ^12^, ^14^, ^15^, ^16^, ^17^, ^18^, ^19^ ^]^ Such chloride‐containing additives have become popular as they do not only influence the morphology of perovskite films,^[^ ^20^, ^21^, ^22^, ^23^ ^]^ but also improve their transport properties and stability.^[^ ^24^, ^25^, ^26^, ^27^, ^28^, ^29^, ^30^, ^31^ ^]^ These changes occur despite the high volatility of the alkylammonium chloride generally leaving only trace amounts of Cl^−^ within the material.^[^ ^32^, ^33^, ^34^ ^]^ While the impact of chlorides has been extensively studied, so far no complete mechanism has been put forward to explain their role in the nucleation and subsequent crystallization.

Here, we elucidate the origin of MACl‐induced vertical crystal orientation by assisting the crystallization of the quasi‐2D perovskite through the addition of excess MACl with an optimized weight ratio *m*_MACl_/*m*_MAI_ of 0.5 (equivalent to a molar ratio of ≈1.17 MACl:MAI). The resulting material does not degrade after exposure to 97% relative humidity (RH) for at least 10 h, which, to our knowledge, is the highest atmospheric humidity perovskites with such a large *n*‐value have been exposed to without degradation. The morphology and crystal structure of the material with and without MACl are analyzed using scanning electron microscopy (SEM), grazing‐incidence small‐ and wide‐angle X‐ray scattering (GISAXS and GIWAXS), and transmission electron microscopy (TEM) techniques to confirm the achieved preferential orientation. Solid‐state nuclear magnetic resonance (ssNMR) of the as‐deposited perovskite films on flexible substrates is measured to assess their local structure and for the first time determine the speciation of Cl^−^ ions within the 2D/3D phase when prepared under identical conditions as the films used in the photovoltaic devices. By interrupting the crystallization process and analyzing the partially crystallized films, we investigate whether the crystallization is initiated at the liquid–air interface in samples fabricated using MACl when DMF is used as a solvent. With this, we formulate a thermodynamic description of the MACl‐assisted crystallization process that can also be generalized to other preferred orientation‐inducing methods, as well as used to design guidelines for future solar cell fabrication.

## 2 Results and Discussion

To investigate the effect of MACl on the perovskite, we first fabricated films and solar cells using the (A′)_2_(MA)*_n_*_−1_Pb*_n_* I_3_ *_n_*_+1_ absorber, where *n* refers to the stoichiometry of the precursor solution used during fabrication and A′ to a large spacer cation. We have chosen *n* = 7 to strike a balance between device stability and performance. Recent research has demonstrated how fluorinating the large organic cation improves its hydrophobicity and consequently the stability of the device, with polyfluorination leading to better stability but also higher formation energies.^[^ ^35^, ^36^, ^37^, ^38^, ^39^, ^40^, ^41^, ^42^ ^]^ Further, fluorination of the meta position of the benzyl group was reported to result in better solubility of the cation compared to the ortho and para position, leading to more crystalline films.^[^ ^43^ ^]^ To avoid high formation energies and poor solubility potentially obscuring the effect of MACl on the nucleation, we therefore chose to implement the monofluorinated 3‐fluorobenzylammonium (3FBA). We establish that the reactivity of 3FBAI is analogous to the parent benzylammonium (BA) iodide by preparing (3FBA)_2_PbI_4_ (*n* = 1) using mechanosynthesis and recording its powder X‐ray diffraction pattern (XRD) pattern (Figure S1, Supporting Information). The Rietveld refinement against the BA analogue, BA_2_PbI_4_ (*Pbca* space group), shows a good overall match, indicating that the iodoplumbate made using 3FBA also has a layered 2D structure.

**Figure 1:**
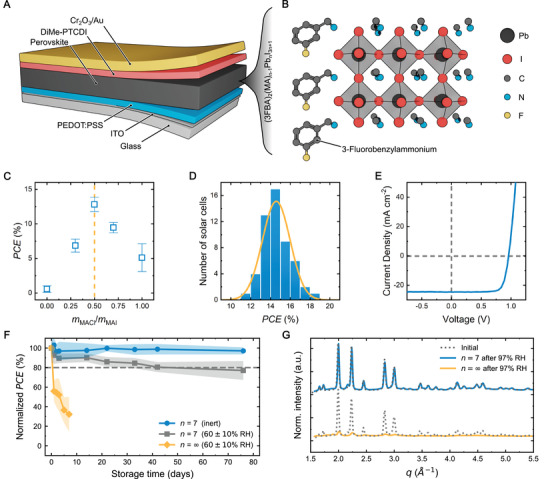
Solar cell characterization and stability. A) Architecture of the solar cell. B) Schematic of the perovskite crystal structure with the incorporated 3FBA organic spacer cation. Hydrogens are omitted for clarity. C) PCE as a function of the mass ratio *m*_MACl_/*m*_MAI_ in the precursor solution. The yellow dashed line indicates the optimized ratio of 0.5. D) Histogram, and E) champion *J*–*V* characteristics of the optimized solar cells with *m*_MACl_/*m*_MAI_ = 0.5. F) Storage stability of the unencapsulated cells containing 3FBA (*n* = 7) kept in the dark either in a N_2_ atmosphere or under atmospheric conditions with a high humidity of 60 ± 10% RH and temperatures of 21 ± 2 °C. Devices without 3FBA (*n* = ∞) are also shown as a reference. The grey dashed line indicates the 80% mark. The error bands correspond to 2 standard deviations (as obtained from 8 devices). G) GIWAXS patterns of perovskite films with and without 3FBA before (dotted lines) and after (solid lines) 10 h of exposure to air with 97% RH. Intensities are normalized to the highest peak of the initial patterns, respectively.

To determine the optimal amount of MACl for photovoltaic performance, we fabricated inverted solar cells consisting of indium‐tin oxide (ITO) as the bottom electrode, poly(3,4‐ethylenedioxythiophene) polystyrene sulfonate (PEDOT:PSS) as the hole‐transport layer (HTL), stochiometric (3FBA)_2_(MA)_6_Pb_7_I_22_ as the photoabsorbing layer, *N*,*N*′‐dimethyl‐3,4,9,10‐perylenetetracarboxylic diimide (DiMe‐PTCDI) as the electron‐transport layer (ETL), and Cr_2_O_3_/Au top contacts (**Figure** **1**A,B). We found that devices made using this perovskite composition on its own result in low photovoltaic performance due to shunts. However, the addition of MACl into the perovskite precursor solution improves the quality of the devices significantly (Figure 1C). Solar cells with an ideal mass ratio of *m*_MACl_/*m*_MAI_ = 0.5 yield average power conversion efficiencies (PCEs) of (14.6 ± 1.4)% from 53 devices with good reproducibility (Figure 1D) after the optimization of the fabrication procedure (Figures S2–S5, Supporting Information). The best device performance of up to 18.5% PCE was achieved with an open‐circuit voltage (*V*_oc_), short circuit current density (*J*_sc_), and fill factor (FF) of 0.97 V, 24.6 mA cm^−2^, and 76.5%, respectively (Figure 1E). Introducing MACl, in combination with the hydrophobic 3FBA cation, also results in the optimized devices exhibiting good stability, showing no noticeable degradation when stored in a nitrogen atmosphere in the dark. Even when continuously exposed to a high humidity of 60% RH, they retain 80% of their initial performance for more than 43 days (Figure 1F and Figure S6, Supporting Information). In contrast, reference devices without the large 3FBA cation (referred to as *n* = ∞, that is, pure MAPbI_3_) degrade significantly after only 1 day of exposure to this humid environment. To investigate if this degradation is mainly caused by the failure of the active perovskite layer, XRD patterns were recorded for films before and after subjecting them to 97% RH for 10 h (Figure 1G and Figure S7A–L, Supporting Information). Under these conditions, the reference perovskite without 3FBA (*n* = ∞) degrades almost entirely, exhibiting a vertically heterogeneous distribution of phases in GIWAXS. Meanwhile, the films containing 3FBA, despite the relatively large *n*‐value of *n* = 7, show no sign of significant degradation and retain the perovskite in its pristine form throughout the bulk, similar to their lower *n* counterparts. To assess if the results also translate to longer timescales, perovskite films with *n* = 7 were also measured while exposed to 80% RH for up to 40 h (Figure S7M, Supporting Information). Even after this prolonged exposure, the diffraction pattern does not show evidence of any significant degradation. Therefore, the observed decrease in PCE of the solar cells can be mainly attributed to degrading charge‐transport layers (CTLs) or changes at the interfaces, and not to the degradation of the perovskite layer.

**Figure 2:**
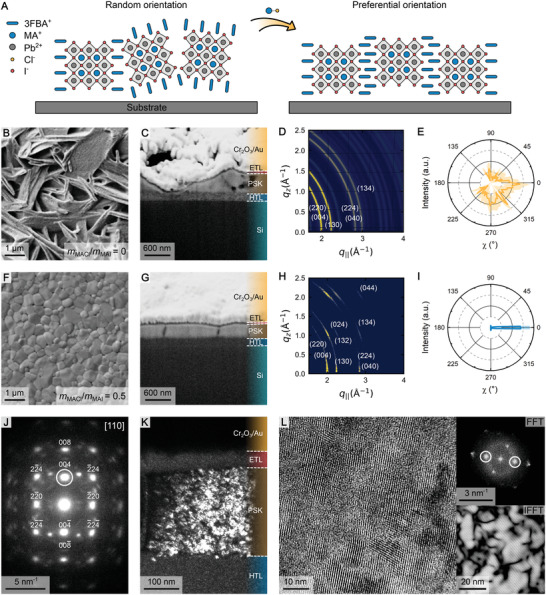
Crystal orientation. A) Schematic of randomly oriented crystallites (left) and crystallites oriented vertically relative to the substrate (right). B) Top and C) cross‐sectional SEM images, D) GIWAXS images (indexed as tetragonal MAPbI_3_, I4cm, #108), and E) peak anisotropy data (at *q* = 1 Å^−1^, corresponding to the (004) and (220) lattice planes) of the scattered X‐ray intensity as a function of the detector angle χ for *n* = 7 perovskite films as grown with *m*_MACl_/*m*_MAI_ = 0 and F–I) *m*_MACl_/*m*_MAI_ = 0.5. J) SAED pattern of a single grain, aligned along the [110] zone axis (indexed as tetragonal MAPbI_3_, (I4/mcm, #140).^[^ ^46^ ^]^ The highlighted condition results in the K) DF TEM image of the cross‐section of a perovskite film fabricated with *m*_MACl_/*m*_MAI_ = 0.5. The bright regions correspond to nanocrystals with the same orientation. L) HRTEM image of the same film providing a closer view of the preferential orientation. The insets show the FFT of the image, with the highlighted reflexes resulting in the reconstructed IFFT image, where domains of similar orientation are shown as bright.

The substantial increase in performance and stability upon using MACl as an additive has been attributed by previous investigations to a significant change in the crystal structure, aligning the organic sheets vertically to the substrate (**Figure** **2**A).^[^ ^11^, ^12^ ^]^ To verify if this is also the case here, first the perovskites grown without MACl were investigated using scanning electron microscopy (SEM). This reveals an irregular needle‐like structure, consistent with previous reports of perovskites grown under stochiometric conditions (Figure 2B,C).^[^ ^44^ ^]^ High‐energy GIWAXS measurements show concentric powder rings characteristic for randomly distributed crystallites (Figure 2D and Supplementary Note 1 in the Supporting Information). This isotropy can be verified by analyzing different diffraction peaks as a function of the azimuthal detector angle χ (Figure 2E and Figure S8A–D, Supporting Information).

However, this behavior changes dramatically when assisting the crystallization with MACl. For the optimized mass ratio of *m*_MACl_/*m*_MAI_ = 0.5, SEM images show much more uniform grains and film thickness, with a Feret's diameter of 500 ± 170 nm as measured from the top view (Figure 2F,G). Aside from lowering the probability of pinhole formation, this improved morphology also reduces the surface area of the film, as well as the number of defects, thus providing fewer sites for water or oxygen to degrade the film. Additionally, the film exhibits an anisotropic diffraction signal, indicating a high degree of preferential orientation of up to 90% compared to the 33% of their counterparts with *m*_MACl_/*m*_MAI_ = 0 (Figure 2H,I and Note S2, Supporting Information). This effect is augmented to 94% by using an antisolvent during film formation, but also occurs without it (Figure S8E–M, Supporting Information). The observed improvement is likely due to the antisolvent washing away excess solvent, starting from the surface of the liquid. As a result, supersaturation occurs faster at the liquid–air interface, slightly augmenting the effect of MACl. Complementary GISAXS data was recorded to access information about the lower *q* range in the reciprocal space (Figure S9, Supporting Information). While the GISAXS images do display a similar preferential orientation to their GIWAXS counterparts, they do not indicate the presence of superstructure phases. To gain further insight into the phase composition of the sample, photoluminescence spectra for films of varying *n*‐value were measured (Figure S10, Supporting Information). While the additional high‐energy bands observed for samples with *n* = 3 and 5 indicate a distribution of low‐*n* phases within the film, no definitive evidence of additional phases is present for *n* = 7. We note, however, that these measurements do not preclude the presence of low‐*n* structures below the detection limit. Therefore, there is likely a broad distribution of *n*‐values with insufficiently coherent crystallographic regions present in the final film.^[^ ^45^ ^]^ The favorable crystal alignment was further verified using our observations from TEM techniques. A representative selected‐area electron diffraction (SAED) pattern collected from a single grain, aligned along the [110] zone axis, reveals a predominant crystal orientation (Figure 2J and Note S3, Supporting Information). The observed crystal structure and measured lattice parameters correspond to tetragonal MAPbI_3_ and are in good agreement with the literature data.^[^ ^46^ ^]^ By choosing one of the diffraction reflexes we could obtain a dark‐field (DF) TEM image, which displays regions of similar orientation as bright (Figure 2K). It turns out that the grain contains numerous similarly oriented nanocrystals separated by differently oriented regions (dark areas). This is further evidenced by a high‐resolution (HR)TEM image, which allows the direct observation of the aligned crystallites with a median size of 10–20 nm (Figure 2L). The fast Fourier transform (FFT) of the image displays preferential orientation, which is visualized by the reconstructed inversed (I)FFT image where the regions of the same selected orientation appear brighter (Figure 2L, inset). Evidently, the presence of MACl in the precursor alters the crystallization process to favor a preferential orientation in the material.

**Figure 3:**
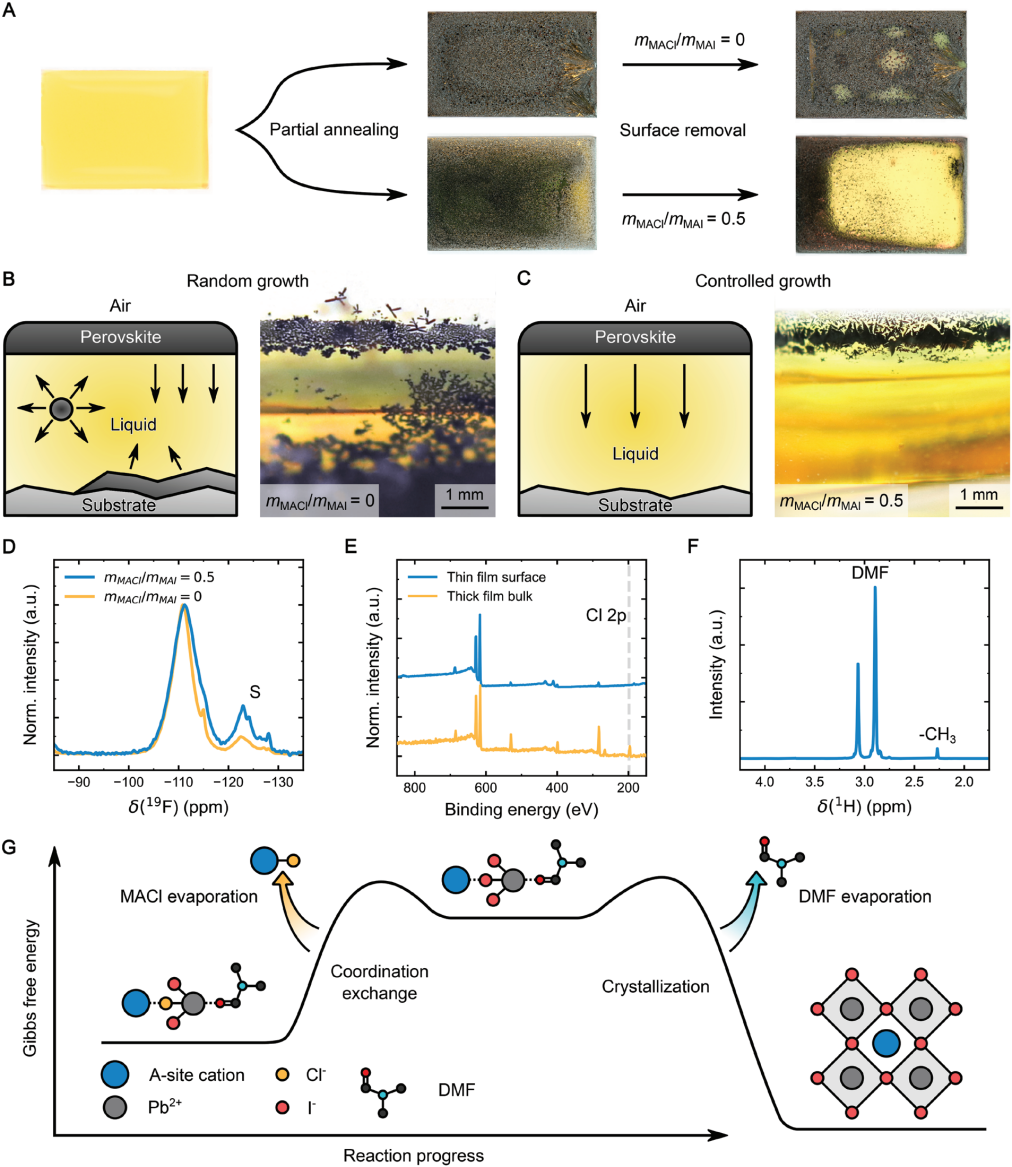
Crystallization mechanism. A) Optical images of the crust removal test. First, the yellow precursor solution (left) is partially annealed until the film turns into the black perovskite phase (middle). Then, the films surface is scraped off (right) to reveal the remaining material underneath. The sub‐surface phase is solid for *m*_MACl_/*m*_MAI_ = 0 (top) and liquid for *m*_MACl_/*m*_MAI_ = 0.5 (bottom). B) Schematics (left) illustrating the crystal growth direction upon nucleation at the liquid–air interface, the liquid–substrate interface, or inside the liquid, as well as optical images (right) of the side view of the precursor solution without and C) with MACl during the initial crystallization step. The solution with the MACl additive has preferential nucleation sites at the liquid–air interface whereas the one without MACl additionally randomly forms crystallites inside the liquid or at the liquid–substrate interface. D) ^19^F solid‐state MAS NMR spectra of perovskite films fabricated on flexible substrates with and without MACl. Background signals from residual perfluorosilane on the substrate are indicated by “S”. E) Selected region of the XPS survey spectra of a regular thin films’ surface (showing no signs of Cl), and of a thick films’ bulk (showing a Cl peak), both grown with *m*_MACl_/*m*_MAI_ = 0.5. The dashed line indicates the location of the Cl2p photoelectron line. F) Solution ^1^H NMR spectrum of the condensed liquid generated from the evaporation of the precursor solution during annealing. G) Illustration of the proposed crystallization mechanism. The ACl·PbI_2_·DMF complex is stabilized through the strong Cl–Pb interaction (left). As heat is introduced into the system, the volatile MACl escapes from the solution, leaving a less stable AI·PbI_2_·DMF complex behind at the liquid–air interface (middle). Since this state is thermodynamically less favorable, nucleation readily occurs as the DMF evaporates to form the perovskite crystal (right).

Previous research proposed that such oriented perovskites originate from the nucleation and subsequent crystallization beginning at the liquid–air interface.^[^ ^2^ ^]^ To investigate if this holds true in the case of MACl‐induced vertical orientation, the drop‐cast yellow precursor solutions with *m*_MACl_/*m*_MAI_ = 0 and 0.5 were partially thermally annealed until the black perovskite phase started to form (**Figure** **3**A). The surface of the films was scraped off using a razor blade. If no MACl is present during the crystallization, visual inspection reveals either a solid yellow phase (possibly corresponding to PbI_2_) or the black perovskite phases beneath the surface. In contrast, despite a similar black appearance, performing the same scraping for films grown with MACl reveals the yellow, non‐crystallized liquid phase beneath a thin solid crust that formed at the liquid–air interface. This effect can be further demonstrated by partially annealing a larger volume of the precursor solution. In this case, tilting the substrate with the resulting films prepared with MACl causes a wave of unannealed precursor solution to ripple underneath the black perovskite crust (Video S1, Supporting Information). Preparing a film this way causes the crust to become too thick for the remaining trapped DMF to escape. Even after annealing the films for >1 h at 100 °C, there is still liquid contained under the perovskite layer. Meanwhile, no residual precursor solution can be observed when performing the same experiment without MACl.

If the nucleation is initiated at the liquid–air interface, crystal growth has a clear preferential orientation compared to nucleation in the isotropic liquid or at the rough liquid–substrate interface (Figure 3B). This crystallization process can be directly observed on a macroscopic scale by starting the crystallization inside a transparent glass vial and observing the cross‐section of the liquid. In the absence of MACl, random crystallization is observed within the liquid, as well as at the liquid–air and liquid–glass interface. In contrast, controlling the nucleation dynamics using MACl constrains the growth to exclusively start at the liquid–air interface (Figure 3C).

One of the important questions related to the role of MACl is whether the chloride ions remain in the film after annealing, and if so, whether they are incorporated into the iodide‐rich perovskite phase. This question has been previously studied in the context of MAPbI_3−_ *_x_* Cl*_x_* using angle‐resolved X‐ray photoelectron spectroscopy (AR‐XPS),^[^ ^24^ ^]^ and X‐ray fluorescence,^[^ ^32^ ^]^ which showed that chlorides are present in annealed films, although those measurements do not provide any chemical information on the phase in which the chlorides are present. More recently, solid‐state NMR has been used to unambiguously show that metastable MAPbI_3−_ *_x_* Cl*_x_* solid solutions do form.^[^ ^33^ ^]^ However, those experiments were carried out on single crystals made without the thermal annealing step used in the fabrication of thin films. To obtain information on local structure and elucidate the speciation chlorides in our materials, we carried out solid‐state magic‐angle‐spinning (MAS) NMR experiments on perovskite films fabricated with and without MACl on flexible parylene C (ParC) substrates (Figure 3D, Figure S11A, and Table S2, Supporting Information). While MAS NMR on perovskite thin films is highly challenging,^[^ ^47^, ^48^ ^]^ here, we take advantage of the fluorinated cation, which allows us to use the highly receptive ^19^F isotope. The ^19^F MAS NMR spectrum of the material without MACl shows a broad (FWHM 4.12 ± 0.06 ppm) peak centered at ‐110 ppm, which is shifted relative to that of neat 3FBAI (‐113 ppm) and corresponds to 3FBA spacer cations within the 2D/3D perovskite (note that the peaks between −120 and −130 ppm correspond to residual perfluorosilane present on the flexible substrate). In the material with MACl, this peak is slightly shifted (−111 ppm) and substantially broadened (FWHM 5.5 ± 0.1 ppm), indicating that the addition of MACl changes the local environment of the spacer cation in the 2D/3D phase. This effect is readily explained by the halide disorder introduced by chlorine incorporation into the iodide‐rich 2D/3D phase. While in the material without MACl the spacer is surrounded only by iodides, the introduction of chlorides into the structure leads to a distribution of local environments whereby the spacer can have a non‐zero number of nearest neighbor chloride ions. The presence of this distribution leads to the experimentally observed peak broadening in the material containing MACl. This result unambiguously evidences that chlorides are incorporated into the 2D/3D perovskite structure. Additionally, GIWAXS and GISAXS patterns reveal new small peaks appearing in samples fabricated using MACl, which we attribute to trace amounts of a phase‐segregated chloride‐rich phase (Figure S11B, Supporting Information).

We also attempted recording ^13^C MAS NMR spectra on the thin film samples, and while we were able to detect MA, the spacer was too dilute to yield signal after 44 h of acquisition at 20 T (Figure S11C and Table S3, Supporting Information). To circumvent this sensitivity limitation, we also prepared the *n* = 1 and *n* = 7 materials using mechanosynthesis and recorded their ^13^C MAS NMR spectra and XRD patterns (Figure S11D,E, Supporting Information). The local environment of 3FBA in these two phases is substantially different than in neat 3FBAI, as evidenced by the shift and splitting of the ^13^C peaks. On the other hand, the local structure of 3FBA in *n* = 7 is similar to that in *n* = 1 with small differences around the —CH_2_— group and in the aromatic ring, indicating that its conformation adapts depending on the number of surrounding 3D slabs. A similar effect has been previously observed in other 2D/3D halide perovskites.^[^ ^49^ ^]^

To estimate the amount of chloride in the film, energy‐dispersive X‐ray spectroscopy (EDX) was conducted in scanning (S)TEM mode to record the general EDX spectrum of the perovskites cross‐section (Figure S11F, Supporting Information). However, the low Cl content and the overlapping of the most intense Cl K series peak with the Pb M series peak rule out reliable quantification. By deconvoluting the Pb and Cl signals, we estimate the trace Cl content in the sample to be <1 at%. To address the challenge of Cl detection, X‐ray photoelectron spectroscopy (XPS) of the perovskite films was performed. While the survey spectrum of the surface does not show any characteristic Cl peaks, measuring the bulk of a roughly 10× thicker film (3500 ± 700 nm) reveals significant amounts (≈7.5 at%) of Cl (Figure 3E and Figure S11G, Supporting Information). This result confirms that the majority of Cl^−^ evaporates from the system during annealing and is consistent with previous reports.^[^ ^32^ ^]^ However, so far it is unclear whether the Cl^−^ can evaporate directly from the solution or only escapes into the gas phase after prolonged heating of the solid perovskite. To clarify this, the composition of the evaporated gas was analyzed by evaporating the precursor solution at the same temperature used during the annealing of the films (100 °C) and letting the generated vapors re‐condense onto a glass slide. Investigating the thus obtained liquid using ^1^H NMR reveals small amounts of methyl groups in the sample (Figure 3F and Figure S12A, Supporting Information). Together, these results directly confirm that some MACl evaporates from the precursor solution before the crystallization of the solid perovskite bulk, while the majority evaporates from the solid phase after prolonged annealing. However, since the crystallization starts from the liquid–air interface, if a thick enough perovskite crust forms between the air and the remaining liquid, some of the Cl^−^ can no longer escape and gets trapped in the bulk. This also explains why residual Cl^−^ is usually only found in significant quantities at the interface of the perovskite and the underlying substrate.^[^ ^24^ ^]^

Because both the solvent and MACl have to evaporate from underneath the already formed crust, it raises the question if this process causes damage to the forming perovskite layer. Microscopy images of films fabricated with *m*_MACl_/*m*_MAI_ = 0.5 display a compact, pinhole‐free surface (Figure S12B, Supporting Information). However, increasing the Cl^−^ content in the solution beyond the optimum ratio to *m*_MACl_/*m*_MAI_ = 1.0 leads to violent evaporation, forming tunnels throughout the film (Figure S12C, Supporting Information). This results in a vast number of pinholes that shunt the device, decreasing performance. Therefore, tuning the MACl ratio is critical to ensure the formation of a continuous active layer.

Combining the observations discussed above, we propose a thermodynamic mechanism of the process leading to highly oriented perovskite films (as illustrated in Figure 3G): Upon adding excess MACl to the precursor solution, the coordination complex PbI_2_·AI·MACl·DMF is formed (where A is the A‐site cation, such as MA, or a larger cation, such as 3FBA). Due to the stronger coordination of MACl to PbI_2_ compared to MAI (resulting from Pb—Cl and Pb—I bond dissociation energies of ≈3.1 and 2.0 eV, respectively),^[^ ^50^, ^51^ ^]^ this intermediate is relatively stable, suppressing the uncontrolled reaction of MAI and PbI_2_.^[^ ^44^ ^]^ However, as heat (Δ) is introduced to the system, since MACl has a higher volatility than MAI,^[^ ^52^, ^53^ ^]^ it readily escapes from solution. Because the evaporation occurs at the liquid–air interface, this process leaves the thermodynamically less stable PbI_2_·AI·DMF complex behind at the surface of the solution. There, the complex aligns itself, likely due to surface tension,^[^ ^2^ ^]^ leading to preferential orientation. As heat continues to be supplied, enough of the high‐boiling‐point, coordinating solvent (such as DMF) also evaporates, allowing the already destabilized PbI_2_·AI·DMF to react, forming the APbI_3_ phase. Note that even if initially only small amounts of MACl evaporate before supersaturation is reached, the metastable intermediates they leave behind still serve as nucleation centers to initiate the subsequent crystallization. This entire process can be summarized in the following 2‐step reaction pathway:

(1) $${\text{PbI}}_{2}\cdot \text{AI}\cdot \text{MACl·DMF}\underset{\Delta }{\;\overset{\text{MACl}\text{​}↑}{\to }}\;{\text{PbI}}_{2}\cdot \text{AI}\cdot \text{DMF}\;\underset{\Delta }{\overset{\text{DMF}\text{​}↑}{\to }}\;{\text{APbI}}_{3}$$

Note that perovskite precursor solutions were reported to be colloidal dispersions rather than real solutions.^[^ ^44^ ^]^ Therefore, the solution complexes are generally more involved compared to the simplified depiction chosen above. Nevertheless, this model can still be used to explain the nucleation reaction as only a small number of molecules need to interact to form the initial nucleation centers from which the subsequent crystallization occurs.

This mechanism can be expanded to other preferential orientation‐inducing methods and used to derive design principles for the development of new additives. First, the additives should have a stabilizing effect on the initial solution complex, preventing uncontrolled crystallization inside the liquid phase or at the liquid–substrate interface (examples of such additives include PbCl_2_ or DMAc). Furthermore, the additives should either be volatile themselves, or form volatile compounds that easily leave the reaction medium to initiate the crystallization at the liquid–air interface (e.g., NH_4_SCN). At the same time, it is critical to keep the amount of the evaporation‐driven, nucleation‐inducing additive low enough to prevent pinholes from forming due to the escaping gas. It should be noted that even at low concentrations, while pinholes may not form in the center of the sample, they may still form at the edges of the perovskite film. This is because as the perovskite crust forms above the liquid, it is easier for the gas to escape at the not‐yet fully formed edges of the film. These may still shunt the device even if they are outside the active area of the solar cell (as defined by the overlap of top and bottom electrode) if a highly conductive CTL is employed.

Additionally, this reaction mechanism is also capable of explaining the effect of fabrication methods that do not rely on additives, such as hot‐casting. Here, the substrate is pre‐heated prior to spin‐coating, increasing solvent evaporation during the spin‐coating step. As a result, the black perovskite phase forms as the sample is still rotating. Since this rapid evaporation occurs at the liquid–air interface, the perovskite nucleates at the liquid surface first, again leading to preferentially aligned crystallites.

Finally, while preferential orientation is of special significance to 2D and quasi‐2D perovskites, controlling nucleation is vital in other cases as well. For example, uncontrolled growth on rough substrates can cause poor film morphology. However, if the nucleation starts at the liquid–air interface, the roughness of the underlying film is almost irrelevant to the formation of the perovskite. This may lead to more defect tolerant, and therefore cheaper, fabrication processes, which are especially relevant for scaling up production with roll‐to‐roll processing on flexible, often plastic, substrates.

## 3 Conclusion

We have fabricated quasi‐2D solar cells by incorporating the large hydrophobic cation 3FBA, greatly improving the resilience of the films against atmospheric humidity. Using the MACl additive, we were able to control the nucleation and subsequent crystallization to facilitate highly textured films. We carried out multimodal characterization of the resulting materials using a combination of long‐range (diffraction) and short‐rage (NMR) structure probes, electron microscopy and X‐ray spectroscopy. Based on these results, we proposed a thermodynamic mechanism leading to the highly oriented films, which can be applied to a wide variety of contexts and fabrication approaches: While the MACl additive initially stabilizes the solution complex, as it heats up and evaporates a thermodynamically less stable intermediary is left behind at the liquid–air interface. As the solvent evaporates and the saturation of the solution increases, this metastable complex reacts first during nucleation, leading to highly aligned crystals due to the clearly defined orientation of the liquids surface.

The explanatory power of this mechanism can also be adapted to describe the fundamental origins of how other established methods cause vertically aligned perovskite crystals. We expect that the understanding of how to control the nucleation and subsequent crystallization of perovskites will enable the rational design of new additives for perovskite optoelectronics research, to facilitate the development of more defect tolerant and stable photoactive materials as the technology transitions from the laboratory to the market.

## 4 Experimental Section

All the materials and methods used are available as part of the Supplementary Information.

## Conflict of Interest

The authors declare no conflict of interest.

## Author Contributions

L.E.L. conceived the project. L.E.L. conducted the preparation and fabrication of the solar cells and thin films, the characterization of their current–density–voltage characteristics (including stability testing), absorbance and the cross‐sectional imaging of the crystallization, microscopy, as well as the analysis of the data. L.E.L. and S.D. performed the experiments scraping of the surface from partially annealed perovskite films and imaging of the thin‐films using scanning‐electron microscopy. K.F. and B.N. conducted the measurements involving X‐ray scattering, including the stability investigations. A.M. and G.H. were responsible for lamellae preparation and investigated the cross‐sections of the solar cells using transmission electron microscopy. A.M. performed HRTEM, (S)TEM energy‐dispersive X‐ray spectroscopy and X‐ray photoelectron spectroscopy measurements. D.J.K. carried out and analyzed the solid‐state nuclear magnetic resonance experiments. L.E.L. prepared the samples to analyze the evaporated gas and H.S. and W.S. conducted the liquid‐phase nuclear magnetic resonance on the samples. L.E.L. and B.H. investigated the thin films using photoluminescence. F.M. performed photothermal deflection spectroscopy to determine the perovskites bandgap. M.C. performed X‐ray photoelectron spectroscopy on the film surface. L.E.L. and M.C.S. investigated the samples using time‐resolved photoluminescence. L.E.L. and B.H. synthesized 3‐fluorobenzylammonium iodide. L.E.L., S.D., and C.P. prepared the flexible substrates. L.E.L., S.D., B.H., C.P., and M.K. designed the figures and wrote the manuscript. All authors contributed to editing the manuscript. M.K., B.N., M.C.S., N.S.S., and W.S. supervised the research.

## Supporting information

Supporting Information

Supplemental Video 1

## Data Availability

The data that support the findings of this study are available from the corresponding author upon reasonable request.

## References

[1] C. Ortiz‐Cervantes , P. Carmona‐Monroy , D. Solis‐Ibarra , ChemSusChem 2019, 12, 1560. 30699237 10.1002/cssc.201802992

[2] A. Z. Chen , M. Shiu , J. H. Ma , M. R. Alpert , D. Zhang , B. J. Foley , D. M. Smilgies , S. H. Lee , J. J. Choi , Nat. Commun. 2018, 9, 1336. 29626205 10.1038/s41467-018-03757-0 PMC5889398

[3] H. Tsai , W. Nie , J. C. Blancon , C. C. Stoumpos , R. Asadpour , B. Harutyunyan , A. J. Neukirch , R. Verduzco , J. J. Crochet , S. Tretiak , L. Pedesseau , J. Even , M. A. Alam , G. Gupta , J. Lou , P. M. Ajayan , M. J. Bedzyk , M. G. Kanatzidis , A. D. Mohite , Nature 2016, 536, 312. 27383783 10.1038/nature18306

[4] Y. Chen , Y. Sun , J. Peng , W. Zhang , X. Su , K. Zheng , T. Pullerits , Z. Liang , Adv. Energy Mater. 2017, 7, 1700162.

[5] X. Zhang , G. Wu , W. Fu , M. Qin , W. Yang , J. Yan , Z. Zhang , X. Lu , H. Chen , Adv. Energy Mater. 2018, 8, 1702498.

[6] X. Zhang , G. Wu , S. Yang , W. Fu , Z. Zhang , C. Chen , W. Liu , J. Yan , W. Yang , H. Chen , Small 2017, 13, 2. 10.1002/smll.201700611 28692766

[7] S. You , X. Xi , X. Zhang , H. Wang , P. Gao , X. Ma , S. Bi , J. Zhang , H. Zhou , Z. Wei , J. Mater. Chem. A 2020, 8, 17756.

[8] L. Cheng , Z. Liu , S. Li , Y. Zhai , X. Wang , Z. Qiao , Q. Xu , K. Meng , Z. Zhu , G. Chen , Angew. Chem. Int. Ed. 2021, 60, 856. 10.1002/anie.202006970 33021033

[9] L. Cheng , K. Meng , Z. Qiao , Y. Zhai , R. Yu , L. Pan , B. Chen , M. Xiao , G. Chen , Adv. Mater. 2022, 34, 2106380. 10.1002/adma.202106380 34750869

[10] C. Li , J. Hu , S. Wang , J. Ren , B. Chen , T. Pan , X. Niu , F. Hao , J. Phys. Chem. Lett. 2021, 12, 4569. 33970641 10.1021/acs.jpclett.1c01074

[11] F. Zheng , C. Zuo , M. Niu , C. Zhou , S. J. Bradley , C. R. Hall , W. Xu , X. Wen , X. Hao , M. Gao , T. A. Smith , K. P. Ghiggino , ACS Appl. Mater. Interfaces 2020, 12, 25980. 32419455 10.1021/acsami.0c05714

[12] K. Odysseas Kosmatos , L. Theofylaktos , E. Giannakaki , D. Deligiannis , M. Konstantakou , T. Stergiopoulos , Energy Environ. Mater. 2019, 2, 79. 10.1039/c9dt01485c 31225556

[13] M. Lv , X. Dong , X. Fang , B. Lin , S. Zhang , J. Ding , N. Yuan , RSC Adv. 2015, 5, 20521.

[14] Q. Jiang , Z. Chu , P. Wang , X. Yang , H. Liu , Y. Wang , Z. Yin , J. Wu , X. Zhang , J. You , Adv. Mater. 2017, 29, 1703852. 10.1002/adma.201703852 29044741

[15] Q. Li , Y. Zhao , R. Fu , W. Zhou , Y. Zhao , X. Liu , D. Yu , Q. Zhao , Adv. Mater. 2018, 30, 1803095. 10.1002/adma.201803095 30141199

[16] E. H. Jung , N. J. Jeon , E. Y. Park , C. S. Moon , T. J. Shin , T. Y. Yang , J. H. Noh , J. Seo , Nature 2019, 567, 511. 30918371 10.1038/s41586-019-1036-3

[17] N. J. Jeon , H. Na , E. H. Jung , T. Y. Yang , Y. G. Lee , G. Kim , H. W. Shin , S. Il Seok , J. Lee , J. Seo , Nat. Energy 2018, 3, 682.

[18] Q. Jiang , Y. Zhao , X. Zhang , X. Yang , Y. Chen , Z. Chu , Q. Ye , X. Li , Z. Yin , J. You , Nat. Photonics 2019, 13, 460.

[19] L. Wang , H. Zhou , J. Hu , B. Huang , M. Sun , B. Dong , G. Zheng , Y. Huang , Y. Chen , L. Li , Z. Xu , N. Li , Z. Liu , Q. Chen , L. D. Sun , C. H. Yan , Science 2019, 363, 265. 30655439 10.1126/science.aau5701

[20] W. L. Tan , Y. Y. Choo , W. Huang , X. Jiao , J. Lu , Y. B. Cheng , C. R. McNeill , ACS Appl. Mater. Interfaces 2019, 11, 39930. 31532193 10.1021/acsami.9b13259

[21] M. I. Dar , N. Arora , P. Gao , S. Ahmad , M. Grätzel , M. K. Nazeeruddin , Nano Lett. 2014, 14, 6991. 25392941 10.1021/nl503279x

[22] Y. Sun , H. Chen , T. Zhang , D. Wang , J. Mater. Sci. 2018, 53, 13976.

[23] B. Lee , T. Hwang , S. Lee , B. Shin , B. Park , Sci. Rep. 2019, 9, 4803 30886329 10.1038/s41598-019-41328-5 PMC6423327

[24] S. Colella , E. Mosconi , G. Pellegrino , A. Alberti , V. L. P. Guerra , S. Masi , A. Listorti , A. Rizzo , G. G. Condorelli , F. De Angelis , G. Gigli , J. Phys. Chem. Lett. 2014, 5, 3532. 26278605 10.1021/jz501869f

[25] V. L. Pool , A. Gold‐Parker , M. D. McGehee , M. F. Toney , Chem. Mater. 2015, 27, 7240.

[26] C. Quarti , E. Mosconi , P. Umari , F. De Angelis , Inorg. Chem. 2017, 56, 74. 27668448 10.1021/acs.inorgchem.6b01681

[27] J. Hieulle , X. Wang , C. Stecker , D. Y. Son , L. Qiu , R. Ohmann , L. K. Ono , A. Mugarza , Y. Yan , Y. Qi , J. Am. Chem. Soc. 2019, 141, 3515. 30646682 10.1021/jacs.8b11210 PMC7156144

[28] A. Jamshaid , Z. Guo , J. Hieulle , C. Stecker , R. Ohmann , L. K. Ono , L. Qiu , G. Tong , W. Yin , Y. Qi , Energy Environ. Sci. 2021, 14, 4541.

[29] J. Yu , Z. Li , C. Kolodziej , S. Kuyuldar , W. S. Warren , C. Burda , M. C. Fischer , J. Chem. Phys. 2019, 151, 234710. 31864238 10.1063/1.5127875

[30] J. Liu , O. V. Prezhdo , J. Phys. Chem. Lett. 2015, 6, 4463. 26505613 10.1021/acs.jpclett.5b02355

[31] J. Chae , Q. Dong , J. Huang , A. Centrone , Nano Lett. 2015, 15, 8114. 26528710 10.1021/acs.nanolett.5b03556 PMC4746708

[32] E. L. Unger , A. R. Bowring , C. J. Tassone , V. L. Pool , A. Gold‐Parker , R. Cheacharoen , K. H. Stone , E. T. Hoke , M. F. Toney , M. D. McGehee , Chem. Mater. 2014, 26, 7158.

[33] W. C. Qiao , J. Yang , W. Dong , G. Yang , Q. Bao , R. Huang , X. L. Wang , Y. F. Yao , NPG Asia Mater. 2020, 12, 68.

[34] D. Prochowicz , R. Runjhun , M. M. Tavakoli , P. Yadav , M. Saski , A. Q. Alanazi , D. J. Kubicki , Z. Kaszkur , S. M. Zakeeruddin , J. Lewiński , M. Grätzel , Chem. Mater. 2019, 31, 1620.

[35] T. Niu , J. Lu , X. Jia , Z. Xu , M. C. Tang , D. Barrit , N. Yuan , J. Ding , X. Zhang , Y. Fan , T. Luo , Y. Zhang , D. M. Smilgies , Z. Liu , A. Amassian , S. Jin , K. Zhao , S. Liu , Nano Lett. 2019, 19, 7181. 31479275 10.1021/acs.nanolett.9b02781

[36] K. T. Cho , Y. Zhang , S. Orlandi , M. Cavazzini , I. Zimmermann , A. Lesch , N. Tabet , G. Pozzi , G. Grancini , M. K. Nazeeruddin , Nano Lett. 2018, 18, 5467. 30134112 10.1021/acs.nanolett.8b01863

[37] H. Yu , F. Xu , C. Li , B. Yuan , T. Liu , Z. Pan , Y. Zhou , B. Cao , Sol. Energy 2021, 221, 583.

[38] L. Wang , Q. Zhou , Z. Zhang , W. Li , X. Wang , Q. Tian , X. Yu , T. Sun , J. Wu , B. Zhang , P. Gao , J Energy Chem 2022, 64, 179.

[39] M. E. F. Bouduban , V. I. E. Queloz , V. M. Caselli , K. T. Cho , A. R. Kirmani , S. Paek , C. Roldan‐Carmona , L. J. Richter , J. E. Moser , T. J. Savenije , M. K. Nazeeruddin , G. Grancini , J. Phys. Chem. Lett. 2019, 10, 5713.31497955 10.1021/acs.jpclett.9b02224

[40] M. A. Hope , T. Nakamura , P. Ahlawat , A. Mishra , M. Cordova , F. Jahanbakhshi , M. Mladenović , R. Runjhun , L. Merten , A. Hinderhofer , B. I. Carlsen , D. J. Kubicki , R. Gershoni‐Poranne , T. Schneeberger , L. C. Carbone , Y. Liu , S. M. Zakeeruddin , J. Lewinski , A. Hagfeldt , F. Schreiber , U. Rothlisberger , M. Grätzel , J. V. Milić , L. Emsley , J. Am. Chem. Soc. 2021, 143, 1529. 33442979 10.1021/jacs.0c11563

[41] Y. Liu , S. Akin , L. Pan , R. Uchida , N. Arora , J. V. Milić , A. Hinderhofer , F. Schreiber , A. R. Uhl , S. M. Zakeeruddin , A. Hagfeldt , M. I. Dar , M. Grätzel , Sci. Adv. 2019, 5, eaaw2543. 31187060 10.1126/sciadv.aaw2543 PMC6555633

[42] X. Lai , W. Li , X. Gu , H. Chen , Y. Zhang , G. Li , R. Zhang , D. Fan , F. He , N. Zheng , J. Yu , R. Chen , A. K. K. Kyaw , X. W. Sun , Chem. Eng. J. 2022, 427, 130949.

[43] G. Yan , G. Sui , W. Chen , K. Su , Y. Feng , B. Zhang , Chem. Mater. 2022, 34, 3346.

[44] K. Yan , M. Long , T. Zhang , Z. Wei , H. Chen , S. Yang , J. Xu , J. Am. Chem. Soc. 2015, 137, 4460. 25780941 10.1021/jacs.5b00321

[45] R. Quintero‐Bermudez , A. Gold‐Parker , A. H. Proppe , R. Munir , Z. Yang , S. O. Kelley , A. Amassian , M. F. Toney , E. H. Sargent , Nat. Mater. 2018, 17, 900. 30202112 10.1038/s41563-018-0154-x

[46] Y. Yamada , T. Yamada , L. Q. Phuong , N. Maruyama , H. Nishimura , A. Wakamiya , Y. Murata , Y. Kanemitsu , J. Am. Chem. Soc. 2015, 137, 10456. 26263192 10.1021/jacs.5b04503

[47] D. J. Kubicki , S. D. Stranks , C. P. Grey , L. Emsley , Nat. Rev. Chem. 2021, 5, 624. 37118421 10.1038/s41570-021-00309-x

[48] M. P. Hanrahan , L. Men , B. A. Rosales , J. Vela , A. J. Rossini , Chem. Mater. 2018, 30, 7005.

[49] J. V. Milić , J. H. Im , D. J. Kubicki , A. Ummadisingu , J. Y. Seo , Y. Li , M. A. Ruiz‐Preciado , M. I. Dar , S. M. Zakeeruddin , L. Emsley , M. Grätzel , Adv. Energy Mater. 2019, 9, 1900284.

[50] W. Kaiser , E. Radicchi , E. Mosconi , A. Kachmar , F. De Angelis , ACS Appl. Energy Mater. 2021, 4, 9827.

[51] Y.‐R. Luo , Comprehensive Handbook of Chemical Bond Energies, CRC Press, Boca Raton, FL, USA 2007.

[52] W. Song , X. Zhang , S. Lammar , W. Qiu , Y. Kuang , B. Ruttens , J. D. Haen , I. Vaesen , T. Conard , Y. Abdulraheem , T. Aernouts , Y. Zhan , J. Poortmans 2022, 14, 27922. 10.1021/acsami.2c05241 35687012

[53] H. Yu , F. Wang , F. Xie , W. Li , J. Chen , N. Zhao , Adv. Funct. Mater. 2014, 24, 7102.

