# Supplementary material for: Elucidating the Origins of High Preferential Crystal Orientation in Quasi‐2D Perovskite Solar Cells
Source: Adv Mater. 2022 Dec 5;35(5):2208061. doi: 10.1002/adma.202208061 (PMC11475220; doi:10.1002/adma.202208061)
Supplement: Supplementary file 1 — Supporting Information [file ADMA-35-2208061-s001.pdf]

ADVANCED MATERIALS

Supporting Information

for *Adv. Mater.,* DOI: 10.1002/adma.202208061

Elucidating the Origins of High Preferential Crystal Orientation in Quasi-2D Perovskite Solar Cells

*Lukas E. Lehner, Stepan Demchyshyn, Kilian Frank, Alexey Minenkov, Dominik J. Kubicki, He Sun, Bekele Hailegnaw, Christoph Putz, Felix Mayr, Munise Cobet, Günter Hesser, Wolfgang Schöfberger, Niyazi Serdar Sariciftci, Markus Clark Scharber, Bert Nickel, and Martin Kaltenbrunner**

Supplementary Information

Elucidating the Origins of High Preferential Orientation in Quasi-2D Perovskite Solar Cells

Lukas E. Lehner[1, 2], Stepan Demchyshyn[1, 2], Kilian Frank[3], Alexey Minenkov[4], Dominik J. Kubicki[5], He Sun[6], Bekele Hailegnaw[1, 2], Christoph Putz[1, 2], Felix Mayr[7], Munise Cobet[7], Günter Hesser[4], Wolfgang Schöfberger[6], Niyazi Serdar Sariciftci[7], Markus Clark Scharber[7], Bert Nickel[3], Martin Kaltenbrunner[1, 2]*

[1]Division of Soft Matter Physics, Institute of Experimental Physics, Johannes Kepler University, Altenberger Straße 69, 4040 Linz, Austria

[2]Soft Materials Lab, Linz Institute of Technology, Johannes Kepler University, Altenberger Straße 69, 4040 Linz, Austria

[3]Soft Condensed Matter Group, Faculty of Physics, Ludwig-Maximilian University, Geschwister-Scholl-Platz 1, Munich, Germany

[4]Center for Surface and Nanoanalytics, Institute of Experimental Physics, Johannes Kepler University, Altenberger Straße 69, 4040 Linz, Austria

[5]Department of Physics, University of Warwick, Coventry, CV4 7AL, United Kingdom

[6]Institute of Organic Chemistry, Johannes Kepler University, Altenberger Str. 69, 4040 Linz, Austria

[7]Linz Institute for Organic Solar Cells (LIOS) and institute for Physical Chemistry, Johannes Kepler University, Altenberger Straße 69, 4040 Linz, Austria

*Corresponding author. Email: martin.kaltenbrunner@jku.at

Materials

Unless otherwise specified, all chemicals were used as received without further purification and are listed in **table S1**.

Table S1: Chemicals. List of all the chemicals used, their supplier, and their country of origin.

Short name	Commercial name, purity	Supplier	Country
3FBAm	3-Fluorobenzylamine, > 98.0 %	TCI	Germany
AA	Acetylacetone, ≥ 99 %	Sigma-Aldrich	Germany
Acetone	Acetone, technical	VWR Chemicals	France
CB	Chlorobenzene, 99.8 % anhydrous	Sigma-Aldrich	U.S.A.
CeO_2	Cerium(IV) oxide powder < 5 nm, 99.9 %	Sigma-Aldrich	Germany
DiMe-PTCDI	N,N'-dimethyl-3,4,9,10-perylentetracarboxylic diimide	Hoechst	Germany
DMF-d7	N,N-Dimethylformamide-d7, ≥ 99.5 at%D	Sigma Aldrich	Germany
DMF	N,N-dimethylformamide, 99.8 % anhydrous	Sigma-Aldrich	U.S.A.
DMSO	Dimethyl sulfoxide, ≥ 99.9 % anhydrous	Sigma-Aldrich	Germany
Epoxy resin	E131 encapsulation epoxy	Ossila	U.K.
Et2O	Diethyl ether	VWR Chemicals	Belguim
EtOH	Ethanol, absolute for analysis	Merck	Germany
HCl	Hydrochloric acid fuming 37wt%, for analysis	Merck	Germany
Hellmanex	Hellmanex III	Hellma Analytics	Germany
HI	Hydroiodic acid 57 wt% in H_2O, 99.95 %	Sigma-Aldrich	U.S.A.
IPA	2-Propanol	VWR Chemicals	France
K_2SO_4	BioXtra, ≥ 99.0 %	Sigma-Aldrich	Germany
LaB_6	NIST SRM660c	NIST	U.S.A.
MACl	Methylammonium chloride, 99.99 %	Greatcell solar	Italy
MAI	Methylammonium iodide, 99.99 %	Greatcell solar	Australia
Parylene C	Parylene C, Dichloro-[2,2]-paracyclophane	Diener electronic	Germany
PbI_2	Lead(II) iodide, 99.9985 %	Alfa Aesar	U.S.A.
PEDOT:PSS	Clevious PH 1000	Heraeus	Germany
Silane	Trichloro(1H,1H,2H,2H-tridecafluoro-n-octyl) silane, > 97.0 %	TCI chemicals	Germany
Tol	Toluene, 99.8 % anhydrous	Sigma-Aldrich	U.S.A.
Zonyl	Zonyl FS-300 Fluorinated Surfactant	Abcr GmbH	Germany

Methods

Synthesis of 3-Fluorobenzylammonium Iodide

3-fluorobenzylamine (25 g) and hydroiodic acid (30 ml) were diluted in ethanol (54 ml) and stirred for 2 h to react in a round bottom flask kept at 0 °C in an ice bath. The solution was then evaporated to obtain a precipitate and washed 7 times with diethyl ether. Remaining excess moisture and hydroiodic acid were removed from the resulting salt *via* freeze drying by submerging the flask in liquid nitrogen for 2 min, followed by evacuating it for 20-30 min. This step was repeated 8 times or until the precipitated powder appeared white.

PEDOT:PSS Preparation

The PEDOT:PSS precursor solution was prepared by mixing PEDOT:PSS (PH1000) dispersion with v/v 5 % DMSO and v/v 0.07 % Zonyl surfactant. The solution was stirred vigorously for 15 min and then allowed to settle in a fridge overnight. Prior to deposition, it was filtered through a 0.45 µm syringe filter (Minisart RC25, Sartorius, Germany).

Perovskite Precursor Preparation

Unless otherwise specified, the precursor solutions for $(3FBA)_2(MA)_{n-1}Pb_nI_{3n+1}$ perovskites were prepared in a nitrogen filled glovebox (H_2O < 0.1 ppm, O_2 < 0.1 ppm) by mixing the stochiometric amounts of 3FBAI (43.2 g), MAI (81.4 g), MACl (40.7 g), and PbI_2 (275.4 g) for $n = 7$ to create 42 wt% solutions in DMF with m_{MACl}/m_{MAI} = 0.5 or 0, respectively. For the solvent engineering optimization, the total volume of the solution was held constant (at 633 µl), and the volume ratio of either AA or DMSO to DMF was varied. To dissolve the salts, they were stirred on a hotplate (70 °C) for 12-16 h. Before deposition, the precursor solution was cooled down to room temperature.

Solar Cell Fabrication

The solar cells were prepared on ITO-coated glass slides. Before deposition of the various layers, the ITO was etched by immersing the substrates in hydrochloric acid for 10 minutes. Subsequently, they were cleaned in an ultrasonic bath sequentially for 30 minutes in Hellmanex solution (2 vol% in water, 60 °C), deionized water (60 °C), acetone (room temperature), and finally isopropanol (room temperature).

A thin layer of highly conductive PEDOT:PSS was then deposited in ambient conditions (on average 22 °C, 55 % RH) by spin coating at 1500 rpm for 45 s (acceleration 720 $rpm\cdot s^{-1}$). The samples were then briefly dried on a hotplate (122 °C) for 5 s, patterned with water-soaked, lint-free clean room paper towels, and subsequently annealed for 15 min at 122 °C. After letting the samples cool down to room temperature, the PEDOT:PSS was washed to remove excess surfactant by spin coating isopropanol on it in a two-step program of 2000 rpm for 2 s (acceleration 1000 $rpm\cdot s^{-1}$) followed by 4000 rpm for 10 s (acceleration 1000 $rpm\cdot s^{-1}$). Afterwards, the samples were again dried on a hotplate for 15 min at 120 °C and then immediately transferred to a nitrogen filled glovebox (H_2O < 0.1 ppm, $O_2 \cong$ 10 ppm).

Unless otherwise stated, the perovskite crystal layer was grown by spin coating the precursor solution on the PEDOT:PSS layer at 4000 rpm for 40 s (accelerating within 1 s) inside the glovebox. 200 µl of chlorobenzene was dropped within 5 s on the spinning film starting 20 s before the termination of the program. The resulting film transformed into the black perovskite phase upon annealing for 35 min at 100 °C. After annealing for 15-20 min, the samples were briefly taken off the hotplate for about 1 min in order to be coarsely patterned using DMF-soaked cotton buds. Note that in this step, only the bottom contacts (i.e. the ITO) were exposed. However, at the edge of the perovskite layer, the PEDOT:PSS was often not fully covered by the crystal, leading to shunts in the device.

To reduce the shunts present in the final device, the samples were patterned a second time, now in atmospheric conditions (on average 22 °C, 55 % RH). This is achieved by first heating the samples again on a hotplate for 1 min at 120 °C, reducing the likelihood of the PEDOT:PSS tearing during the following. A cotton bud was slightly moisturized by tapping it onto a water-soaked paper towel and then used to pattern the edge of both the perovskite and PEDOT:PSS

layer of the still-hot sample simultaneously. Excess water was then removed by putting the sample back on the hotplate for 5 min at 120 °C.

For the ETL, DiMe-PTCDI was first vacuum-sublimated for purification and then a thin layer (75 nm) was deposited on top of the perovskite absorber using physical vapor deposition. The rate of evaporation was held at 0.2 Å s^{-1} for the first 10 nm and subsequently increased to 0.5 Å s^{-1}. During this process, the temperature of the crucible ranged from 280-310 °C, while the sample holder was water cooled to avoid damage to the devices. The evaporation was initiated at a pressure of $2.5{\cdot}10^{-6}$ Torr.

Finally, the top contacts were evaporated in a nitrogen filled glovebox (H_2O < 0.1 ppm, O_2 < 0.1 ppm) through a shadow mask (final pixel area 0.18 mm). First, a Cr_2O_3 layer was formed by evaporating Cr (10 nm) at 0.1 Å s^{-1}. Then, Au (100 nm) was deposited at a rate of 0.1 Å s^{-1} for the first 10 nm, followed by 1.0 Å s^{-1} for the remaining 90 nm. The evaporation was initiated at a pressure of $5{\cdot}10^{-6}$ mbar.

Mechanosynthesis

Samples with stoichiometries corresponding to n = 1 and n = 7 were made as bulk powders using mechanosynthesis (grinding in the solid state) without the use of solvents, following the previously reported protocol.[1,2] The used precursors were 3FBAI (152 mg, 0.6 mmol) and PbI_2 (138 mg, 0.3 mmol) for n = 1 and 3FBAI (51 mg, 0.2 mmol), MAI (95 mg, 0.6 mmol) and PbI_2 (323 mg, 0.7 mmol) for n = 7. They were weighed out (± 1 mg) into an Eppendorf vial (2 mL) containing a stainless-steel ball (4 mm diameter) under argon. After grinding the material in an electric ball mill (Retsch MM-400) for 30 min at a vibration frequency of 25 Hz, the resulting powder was transferred into glass vials and annealed at 100 °C for 5 min to remove grinding-induced defects.

Current Density-Voltage Measurements

Photocurrent density-voltage (J-V) characteristics were recorded under AM1.5 global spectrum irradiation from a 1600 W xenon light source. The emitted light intensity (100 ± 2 mW cm^{-2}) was calibrated using a commercial Si reference diode (Si-01TC, Ingenieurbüro Mencke & Tegtmeyer, Germany). Two-point measurements were performed by contacting the ITO on two opposite sides of the substrate (and at a single point on the top metal contact), connecting the device to a Keithley-2400-LV source meter operated using LabVIEW software. Unless stated otherwise, J-V sweeps were then conducted from 1.5 V to -1 V (reverse bias) with a voltage step of 0.05 V. Since some devices demonstrated performance improvements upon either light-soaking or the application of forward bias, each pixel was measured several times in reverse, forward and again reverse bias over the span of 15 min. The best resulting performance (determined by the highest respective PCE) was reported.

Storage Stability Testing

Shelf-life investigations were conducted by storing as-prepared, unencapsulated solar cells in the dark either inside a nitrogen-filled glovebox (H_2O < 0.1 ppm and O_2 < 0.1 ppm) or at atmospheric conditions (on average 21 ± 2 °C, 60 ± 10 % RH). Current density-voltage characteristics were recorded after transferring the devices into a nitrogen-filled glovebox (as described above). Reported efficiencies of the devices stored in a humid atmosphere were obtained after light-soaking for approximately 15-30 min, during which the performance continually improved.

X-Ray Diffraction

Unless otherwise specified, X-ray diffraction data were recorded at beamline P07 (PETRAIII, DESY).[3] We use an X-ray energy of 103.6 keV (λ = 0.1197 Å). For simultaneous recording of small- and wide-angle data, two Dexela 4343CT detectors (2880x2880 pixels, 150x150 μm^2 pixel size, Varex Imaging) at 462.6 cm and 77.0 cm distance from the sample were used, each covering one quadrant of the diffraction pattern. The data shown in Figure 2H and Figure S8B were obtained with 103.2 keV, 463.5 cm and 75.7 cm distance, respectively. All detector geometries were calibrated using Cerium(IV) oxide powder.

Samples were prepared on 25.4x25.4 mm^2 ITO-coated glass substrates as shown in Figure 1A, without DiMe-PTCDI and Cr_2O_3/Au layers. The samples were mounted horizontally on a surface diffractometer (Huber Diffraktionstechnik) and aligned parallel to the microfocused X-ray beam (2x30 μm^2). For the measurement, we adjusted a grazing incident angle of $\alpha_i \leq 0.025$ °. The footprint of the X-ray beam was completely within the sample. The X-ray penetration depth was tuned by the incident angle. The exposure time per dataset was 0.5 s.

For stability testing under humid atmosphere, a saturated K_2SO_4 salt solution was prepared by gradual addition of salt to deionized water (RephiLe Purist UV) under vigorous stirring. At a temperature of 25 °C, K_2SO_4 salt provides a humid atmosphere of 97 ± 1 % relative humidity.[4] The samples were stored in air-tight bags sealed inside a nitrogen-filled glovebox until the initial diffraction measurement. To investigate the effect of humidity on the Perovskite structure, each sample was then stored in a 50 ml centrifuge tube together with the salt solution (5 ml) in an open vial. For this purpose, a highly hydrophobic vial made from cyclic olefin copolymer with large surface area (6 cm^2) was used. After 10 h, the tube was opened and the sample was measured again.

Measurements of Perovskite texture were carried out at beamline P21.1 (PETRAIII, DESY) using an X-ray energy of 101.6 keV (λ = 0.122 Å) and a Perkin Elmer XRD1621 flat panel detector (2048x2048 pixels, 200x200 μm^2 pixel size). The detector was placed at 202.6 cm from the sample. Calibration of the detector position was performed using a LaB6 powder standard (NIST SRM660c). The sample was aligned parallel to the X-ray beam (1x1 mm^2). For the measurement, we used zero incidence angle 0 ± 0.1 °. Here, the footprint of the beam always covered the full sample length. The exposure time was 1 s.

Data were transformed to intensity vs. scattering vector $q = \frac{4\pi}{\lambda}\sin(\theta)$ and azimuthal angle χ, 2θ being the scattering angle, using the pyFAI package for python 3.6.9.[5] χ is the azimuthal angle on the detector, with 0 ° corresponding to the direction along the surface normal of the sample.

For the Rietveld refinement, the pattern of the mechanosynthesized *n* = 1 film was recorded on a XRD Panalytical X-Pert Pro MPD instrument equipped with a focusing Johanson monochromator on the incident beam optics, yielding high-resolution pure Cu Kα1 radiation (1.5406 Å).

Scanning Electron Microscopy

Images were collected using a Zeiss CrossBeam 1540 XB SEM (Zeiss, Germany) at 3 keV acceleration voltage, applying a secondary electron detector. The analyzed samples were prepared by growing the respective perovskite thin films on PEDOT:PSS coated ITO substrates (as described above).

Transmission Electron Microscopy

Imaging was carried out in a JEOL JEM-2200FS (JEOL, Japan) operated at an acceleration voltage of 200 kV. The TEM is equipped with an in-column Ω-filter and a TemCam-XF416 (TVIPS,

Germany) CMOS-based camera. Snapshots were recorded applying zero-loss filtering. (HR)TEM data processing was done with Gatan Microscopy Suite and JEMS simulation software. The specimens were prepared in cross-sectional geometry via focused ion beam (FIB) milling (CrossBeam 1540 XB). The FIB was operated at an acceleration voltage of 30 kV and 5 kV for sample lift-out and final thinning, respectively.

Crust-Removal Tests

As-prepared precursor solutions (with a mass ratio of either m_{MACl}/m_{MAI} = 0 or 0.5) were dropped onto oxygen-plasma treated glass slides and thermally annealed in ambient conditions at 100 °C. As soon as the surface turned dark, forming the black perovskite phase, the slides were taken off the hotplate and the surface was scrapped off using a razor blade.[6] To ensure comparability, both investigated solutions were annealed for the same amount of time.

Observation of the Initial Crystallization

To directly observe the initial stages of crystallization cross-sectionally through the liquid, the precursor solution (with a mass ratio of either m_{MACl}/m_{MAI} = 0 or 0.5) was drop cast into a cylindrical glass vial. The top of the vial was cut off to avoid evaporating solvent to condense and flow back down into the solution in the following. To induce supersaturation, the vial was then heated to 100 °C until the black perovskite phase started to form. The sideways view of the vial then reveals the air/liquid/substrate cross-section with crystals growing either at the liquid-air interface, inside the liquid or at the substrate-liquid interface. To ensure comparability, both investigated solutions were annealed for the same amount of time.

Solid-State Nuclear Magnetic Resonance (NMR) Spectroscopy

To measure solid-state Magic Angle Spinning (MAS) NMR on intact perovskite films, the samples were fabricated on flexible ParC substrates instead of glass to enable placing them into 3.2 mm NMR rotors without damaging the films. After cleaning the glass substrates (as described above), they were activated with O_2 plasma for 10 min at 100 W. Immediately afterwards, they were placed inside a closed Petri dish with several drops of perfluorinated silane (Trichloro(1H,1H,2H,2H-tridecafluoro-n-octyl) silane) next to them to prepare a release layer for the parylene, facilitating its later separation from the glass substrate. Surface silanization occurred via vapor phase and was complete after 30 min. Excess silane was removed by washing the glass sequentially in an ultrasonic bath at room temperature using acetone and isopropanol for 20 min, respectively. A 1-2 µm thick flexible ParC film was deposited using a parylene coating system (Parylene P6, Diener Electronic, Germany). The subsequent deposition of the PEDOT:PSS/perovskite layers was performed as described above. Before the measurements, the flexible samples were peeled off the glass substrates and packed into a 3.2 mm zirconia rotor (12 films for each perovskite composition in total). Solid-state MAS NMR spectra of ^{19}F (470.6 MHz) were recorded on a Bruker Avance III 11.7 T spectrometer equipped with a 3.2 mm MAS probe using 83 kHz RF field amplitude. ^{19}F chemical shifts were referenced to the ^{13}C chemical shift of solid adamantane (38.48 ppm for the CH_2 signal) using the ratio of the gyromagnetic ratios in accordance with the IUPAC recommendation. ^{13}C MAS and ^{1}H-^{13}C CP MAS spectra were recorded on a Bruker Avance Neo 20 T (213.8 MHz, thin film samples) or Bruker Avance III 11.7 T (125.8 MHz, samples made by mechanosynthesis) spectrometer equipped with a 3.2 mm CPMAS probe and referenced to solid adamantine. 75-82 kHz ^{1}H decoupling was used. Recycle delays of 3-4 s (^{19}F), 10 s (^{13}C, echo) and 1-7 s (^{1}H-^{13}C CP) were used (see Table S2 and S3 for further acquisition and processing parameters). The rotors were spun using dry nitrogen.

Energy-Dispersive X-Ray Spectroscopy

EDX analysis was performed in scanning (S)TEM mode for qualitative elemental characterization of the specimen's cross-section with a detector from Oxford Instruments (UK). The data were processed with dedicated Aztec software.

X-Ray Photoelectron Spectroscopy

XPS measurements were performed using a ThetaProbe system (Thermo Scientific, UK). The specimens were probed with monochromated Al K_α X-Ray radiation (1486.6 eV) focused into a spot of 100 µm in diameter to ensure a good signal-to-noise ratio. Survey spectra were acquired with a pass energy of 200 eV and a binding energy (BE) step of 1 eV, whereas for the high-resolution spectra, a pass energy of 20 eV with a BE of 0.05 eV was utilized. A dual flood gun was used to compensate for charges accumulated on the surface. The measured spectra were corrected with respect to the C1s peak of the adventitious carbon at 285.0 eV. The spectra were evaluated using the Avantage software package from the device manufacturer. Considering the thickness of the perovskite layer and the XPS spot size, thicker perovskite films had to be used for the bulk analysis. By spin-coating the perovskite precursor solution at 660 rpm for 10 s (acceleration of 120 rpm·s^{-1}), a thickness of about 3500 nm was achieved. To further increase the investigated area, the samples were prepared via ultra-low-angle (≤ 2 °) microtomy (ULAM) with an Ultracut UCT system (Leica, Germany).

Liquid-Phase Nuclear-Magnetic Resonance (NMR) Spectroscopy

To investigate the composition of the gas evaporating during the annealing process, the precursor solution was prepared using deuterated DMF (≥ 99.5 at% D) to reduce the DMF signal in the subsequent NMR measurements. A vial containing the as-prepared solution was covered using a glass slide and heated to 100 °C for about 20 minutes, until enough of the evaporated gas has condensed on the glass cover. Note that during this time, the precursor solution remained entirely liquid, with no black perovskite phase forming anywhere within the vial to ensure that the evaporation of any organic species did not occur from the solid perovskite. This precipitate was then recovered by washing it off using deuterated DMF (DMF-d7). ^{1}H NMR spectra (500 MHz) of the sample were recorded on a Bruker DRX 500 MHz spectrometer.

Photoluminescence Measurements

Photoluminescence spectra were obtained by illuminating the samples with 488 nm light from a 3 mW laser source (OBIS 488 nm, Coherent, U.S.A.) with a spot size of 0.2 cm^{-2}. Light emitted from the perovskites was collected by a monochromator (Shamrock 303-i, Andor Technology, Northern Ireland) equipped with a CCD camera (iDus 420, Andor Technology, Northern Ireland). Measured perovskite films were grown using chlorobenzene as anti-solvent on PEDOT:PSS coated glass slides (cleaning and spin-coating as described for the ITO-coated glass slides above). Investigated samples were oriented such that both the impinging light and the detector faced the same side of the sample (either the glass or perovskite-coated side).

Time-Resolved Photoluminescence Measurements

Time-resolved photoluminescence measurements were performed using a time-correlated-single-photon-counting setup (SPC 150, PMC-100-1 cooled photomultiplier, Becker & Hickel GmbH, Germany) and a supercontinuum laser (SuperK FIANIUM FIU-15, NKT Photonics,

Denmark) equipped with a pulse picker and a wavelength selection unit (LLTF CONTRAST VIS, Photon etc., Canada) for excitation. The laser delivered 5 ps pluses and measurements were performed at an excitation fluence of 0.1 nJ cm^{-2}.

Absorbance and Bandgap Measurements

Absorbance spectra of glass/ITO/PEDOT:PSS/perovskite films were recorded using a spectrophotometer (LAMBDA 1050 UV/Vis Spectrophotometer, Perkin Elmer, U.S.A.). A glass/ITO/PEDOT:PSS thin film was used as a reference.

Photothermal deflection spectroscopy (PDS) measurements were performed on a custom-built setup. Excitation light was provided by a supercontinuum white light laser (SuperK EXTREME EXB-6, NKT Photonics, Denmark) coupled to an acousto-optic tunable filter (SuperK Select, NKT Photonics, Denmark). This setup allows wavelength selection in the range of 450 to 1100 nm with an excitation linewidth (FWHM) of ca. 3 to 6 nm. The excitation light was modulated at a frequency of 1.7 Hz by a mechanical chopper. An optical fiber and a set of lenses was used to focus the light onto the thin film sample. After mounting on a custom-made sample holder, the sample was immersed into the transparent, inert deflection medium (perfluoro(methyldecalin), Flutec PP9, F2 Chemicals) in a 10 mm cuvette (Hellma OS). The diameter of the light spot on the sample was approximately 350 µm. During measurements, a beam splitter and Si photodiode were used to monitor the intensity of the excitation light. A second laser beam (probe beam) from a fiber coupled HeNe laser (K-Cube KLS635, Thorlabs) was passed through the deflection medium at the height of the excitation light spot on the sample, parallel to the thin film surface and aligned as close to the sample as possible without grazing. A position sensing detector (PSD, PDQ80A, Thorlabs) was used to measure the deflection of the probe beam due to heating of the deflection medium via absorption of excitation light by the sample. Lock-in amplifiers (Stanford Research Systems) were used to measure the probe beam deflection signal and the signal from the Si photodiode monitoring the excitation light intensity. A digital multimeter (Keithley 2700) was used to measure the total probe beam intensity on the position sensing detector during measurements. The deflection signal was obtained by correcting the raw deflection signal measured on the PSD with the excitation light intensity and total probe beam intensity signals.

Optical bandgaps were determined assuming that the energy-dependent absorbance $A(\mathrm{h}\nu)$ follows the linear relationship

$$(\mathrm{h}\nu A(\mathrm{h}\nu))^2 = C(\mathrm{h}\nu - E_g)$$

For a direct band gap, where C is some constant, E_g is the optical bandgap, h is Planck's constant and ν is the photon frequency. The bandgaps were obtained by plotting $(\mathrm{h}\nu A(\mathrm{h}\nu))^2$ as a function of the photon energy $\mathrm{h}\nu$, and extrapolation the linear region of the plot of the absorption edge with a linear fit to obtain the intersection with the *x*-axis.[8]

Supplementary Discussion

Supplementary Note 1: XRD Discussion

We recorded XRD data at a grazing incidence angle (GISAXS and GIWAXS) to control the penetration depth of the X-ray beam into the film. We assume a smooth layer of $(3FBA)_2(MA)_{n-1}Pb_nI_{3n+1}$ (n = 7) as the sample and a density of 4.1 g/cm^3, similar to $MAPbI_3$. We use the following expressions for the real and imaginary parts of the refractive index:

$$n = 1 - \delta + \mathrm{i}\beta$$

$$\delta = \frac{N_\mathrm{A} r_0}{2\pi}\frac{\rho\lambda^2 f_1}{M_\mathrm{a}}$$

$$\beta = \frac{N_\mathrm{A} r_0}{2\pi}\frac{\rho\lambda^2 f_2}{M_\mathrm{a}}$$

N_A is the Avogadro constant, r_0 is the classical electron radius, ρ is the material density, λ is the X-ray wavelength, f_1, f_2 and M_a are the atomic form factors and atomic weights of the respective elements. These values were obtained from the NIST X-ray form factor, attenuation and scattering tables and averaged according to the stoichiometry.[9]
As a result, we obtain a value of

$$\alpha_\mathrm{c} = \sqrt{2\delta} = 0.021\,°$$

for the critical angle. We then choose specific incidence angles α_i below and above the critical angle to probe the top and bulk of the film, respectively. The penetration depth Λ_1 of the X-ray beam can be calculated as follows:[10]

$$\Lambda_1^{-1} = \sqrt{2}\frac{2\pi}{\lambda}\left(\sqrt{(\alpha_\mathrm{i}^2 - \alpha_\mathrm{c}^2)^2 + 4\beta^2} + \alpha_\mathrm{c}^2 - \alpha_\mathrm{i}^2\right)^{\frac{1}{2}}$$

For the data shown in **Figure 2D** and **H** we therefore obtain a penetration depth ≤ 110 nm, completely within the Perovskite film. In **Figure S6** the incidence angles of 0.01 ° and 0.025 ° correspond to penetration depths of ca. 3 nm and > 230 nm. Here it must be considered that degradation increased the roughness of the films, which disturbs the grazing incidence condition and leads to larger penetration depth.

Supplementary Note 2: Degree of Preferential Orientation

To quantify the relative difference in preferential orientation in our samples, we define the preferential degree of orientation γ of a specific diffraction peak as follows:

$$\gamma := \frac{1}{\Delta\chi}\int_{\chi_0}^{\chi_1}\left(1 - \frac{I(\chi)}{\max[I(\chi)]}\right)\mathrm{d}\chi$$

Where $I(\chi)$ is the scattered X-ray intensity at a specific peak position q in reciprocal space as a function of the X-ray azimuthal detector angle χ, as measured around the direct beam, and $\Delta\chi = \chi_1 - \chi_0$ is the measured range. Note that this definition is meaningful if $(\Delta\chi\text{-}1\,°)/\Delta\chi \sim 1$. If this condition is fulfilled, then the ideal, perfectly anisotropic signal given by the Dirac delta distribution $\delta(\chi - \chi_\mathrm{peak})$ yields unity:

$$\gamma_\mathrm{ideal} = \frac{1}{\Delta\chi}\int_{\chi_0}^{\chi_1}\left(1 - \delta(\chi - \chi_\mathrm{peak})\right)\mathrm{d}\chi = \frac{\Delta\chi - 1\,°}{\Delta\chi} \approx 1$$

On the other hand, for a perfectly isotropic signal $I(\chi) = \text{const}$ and thus the integrand vanishes, leading to $\gamma_{\text{isotropic}} = 0$. Therefore, under the stated assumptions, $100 \cdot \gamma$ can be interpreted as the degree of preferential orientation in percent.
Note that since $I(\chi)$ for $m_{MACl}/m_{MAI} = 0$ is quite isotropic and therefore noisy, the intensity was first fit using a broad Gaussian to obtain meaningful results. In summary this leads to γ = 33 %, 90 %, and 94 % for samples grown with m_{MACl}/m_{MAI} = 0, 0.5 and 0.5 with antisolvent-assisted film formation, respectively.

Supplementary Note 3: TEM Discussion

The SAED pattern was recorded from a region that slightly exceeded the size of the investigated grain. Therefore, the presented pattern in **Figure 2J** can contain additional reflexes from neighboring grains. Note that the grain in **Figures 2K** and **L** qualitatively appears to have a lower degree of preferential orientation compared to the 94 % obtained from the XRD data. However, these measurements can not be directly compared for two reasons. First, the X-ray measurement averages over a large area, while the TEM only observes a single grain and is therefore less statistically significant. Secondly, the samples for TEM are thin lamellae that are cut out of the perovskite using a FIB. It is possible that this process induces slight changes in the texture of the sample compared to the much less invasive XRD measurements. However, no beam damage was observed after repeated exposure. Regardless, the TEM measurements are only representative of a single grain, not the entire bulk.

Table S2: Details of the ^{19}F NMR experiments. ^{19}F MAS NMR acquisition and processing parameters for the thin film samples shown in Figure 3D of the main text.

Composition	Pulse sequence	^{19}F T_1 [s]	Recycle delay [s]	# Of scans	Magnetic field [T]	MAS rate [kHz]	Apodization [Hz]
Neat 3FBAI	Hahn echo	424	350	80	11.7	20	0
m_{MACl}/m_{MAI} = 0 (12 thin films)	Hahn echo	2.4	3	30513	11.7	20	50
m_{MACl}/m_{MAI} = 0.5 (12 thin films)	Hahn echo	2.7	4	17049	11.7	20	100

Table S3: Details of the ^{13}C NMR experiments. ^{13}C MAS NMR acquisition and processing parameters for the thin film and mechanosynthesized samples shown in supplementary figures S10C and S10D.

Composition	Pulse sequence	^{1}H T_1 [s]	Recycle delay [s]	# Of scans	Magnetic field [T]	MAS rate [kHz]	Apodization [Hz]
Neat 3FBAI	CP	0.6 (measured at 11.7 T)	1.5	3382	20.0	20	0
m_{MACl}/m_{MAI} = 0 (12 thin films)	CP	5.2	7	6729	20.0	20	100
m_{MACl}/m_{MAI} = 0 (12 thin films)	echo	5.2	10	15904	20.0	20	100
$(3FBA)_2PbI_4$ (n = 1, mechano-synthesis)	CP	1.2	1.5	1653	11.7	20	10
$(3FBA)_2(MA)_6Pb_7I_{20}$ (n = 7, mechano-synthesis)	CP	2, 10 (bi-exponential)	5	4096	11.7	20	50

Supplementary Figures

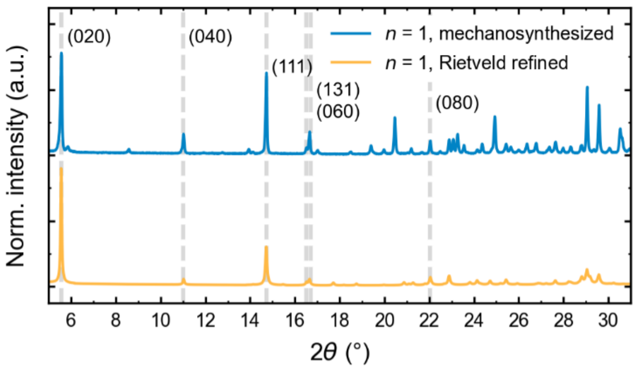

Figure S1: Rietveld refinement. Powder XRD pattern of the neat *n* = 1 $3FBA_2PbI_4$ perovskite as fabricated using mechanosynthesis. To establish similar reactivity to previously used spacers, the structure was Rietveld refined against the benzylammonium (BA) analogue BA_2PbI_4.

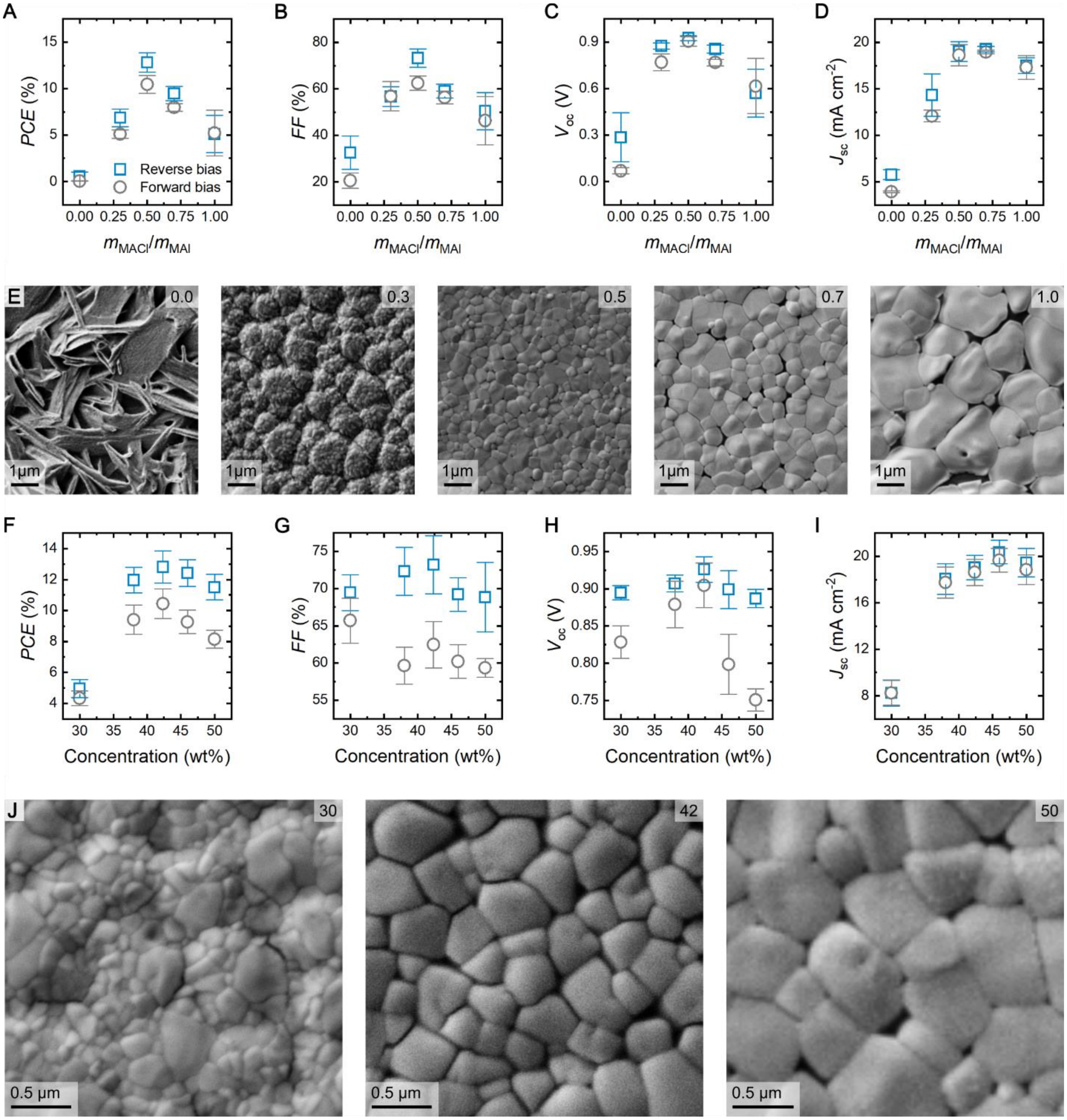

Figure S2: Precursor composition optimization. (**A**) *PCE*, (**B**) *FF*, (**C**) V_{oc} and (**D**) J_{sc} of solar cells fabricated with $n = 7$ and varying amounts of MACl in the precursor solution. (**E**) SEM images of the surface of perovskite films grown with different m_{MACl}/m_{MAI} mass ratios as indicated in the top right of the respective panel. (**F**) *PCE*, (**G**) *FF*, (**H**) V_{oc} and (**I**) J_{sc} of devices created from precursor solutions of different concentrations and (**J**) SEM images of the corresponding perovskite films (the concentration in wt% is given in the top right).

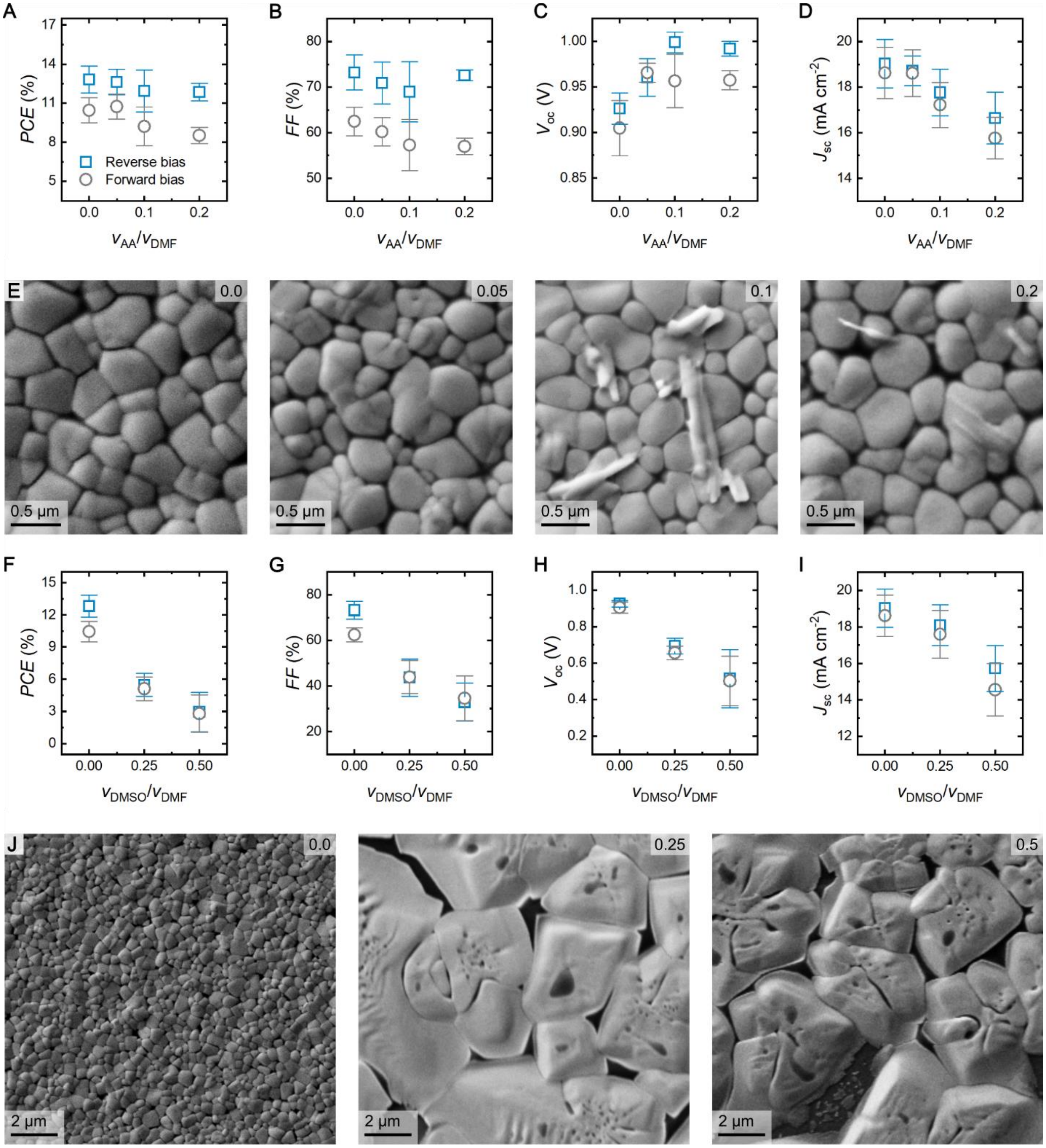

Figure S3: Solvent engineering. (**A**) *PCE*, (**B**) *FF*, (**C**) V_{oc} and (**D**) J_{sc} of solar cells fabricated from precursor solutions where some of the of DMF was replaced by acetylacetone (AA). (**E**) SEM images of the surface of perovskite films grown with different v_{AA}/v_{DMF} volume ratios as indicated in the top right of the respective panel. (**F**) *PCE*, (**G**) *FF*, (**H**) V_{oc} and (**I**) J_{sc} of devices created from precursor solutions containing dimethyl sulfoxide (DMSO) and (**J**) SEM images of the corresponding perovskite films (the volume ratio v_{DMSO}/v_{DMF} is given in the top right).

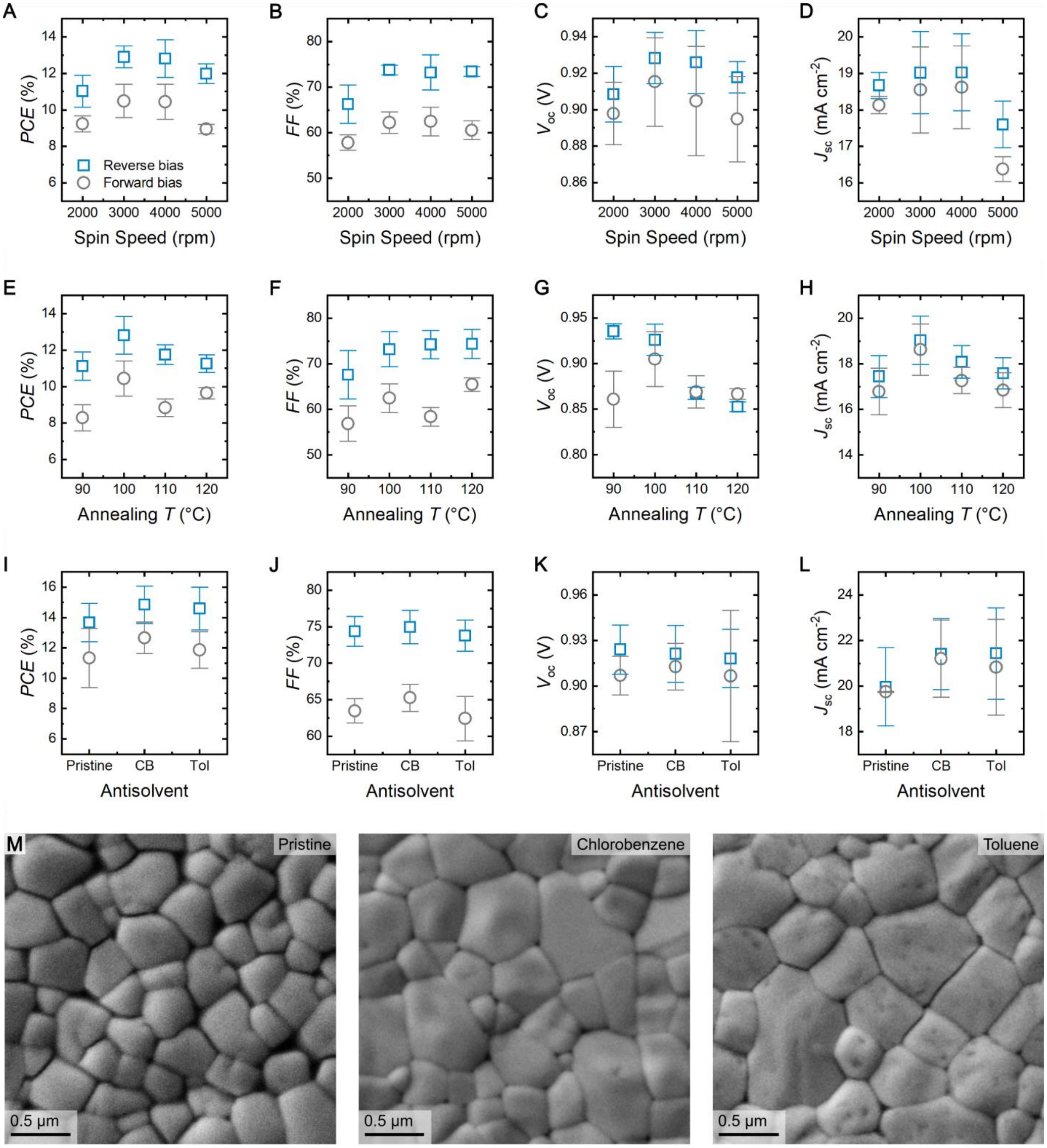

Figure S4: Fabrication optimization. (**A**) *PCE*, (**B**) *FF*, (**C**) V_{oc} and (**D**) J_{sc} of solar cells fabricated from perovskite films deposited at varying spin-coating speeds. (**E**) *PCE*, (**F**) *FF*, (**G**) V_{oc} and (**H**) J_{sc} of devices made using perovskite films annealed at different temperatures *T*. (**I**) *PCE*, (**J**) *FF*, (**K**) V_{oc} and (**L**) J_{sc} of solar cells created either without (pristine) or with the antisolvents chlorobenzene (CB) or toluene (Tol) during the deposition of the perovskite layer and (**M**) SEM images of the corresponding perovskite films (as indicated in the top right of the corresponding panel).

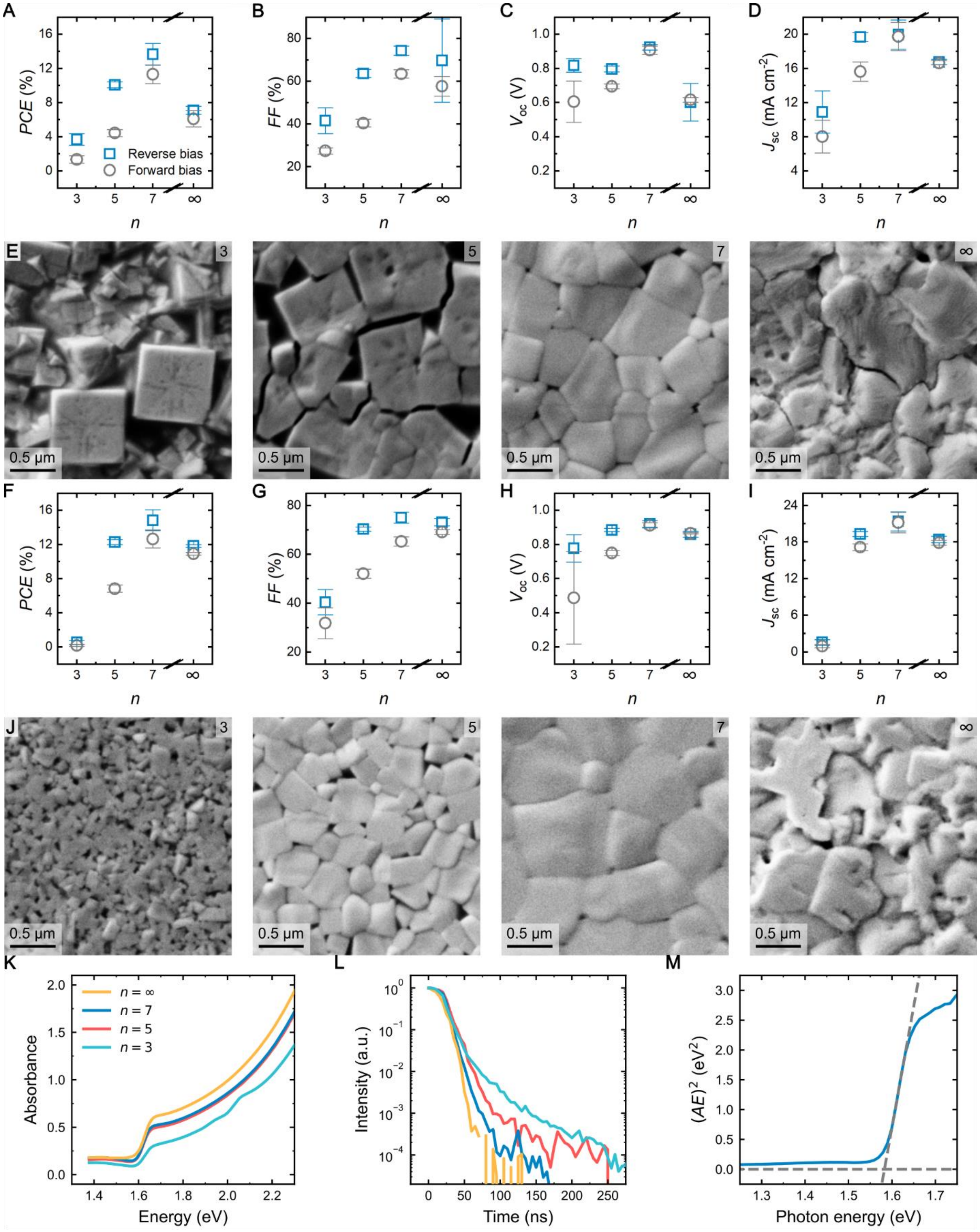

Figure S5: N value optimization. (**A**) *PCE*, (**B**) *FF*, (**C**) V_{oc} and (**D**) J_{sc} of solar cells fabricated with varying *n* value of the perovskite active layer $(3FBA)_2(MA)_{n-1}Pb_nI_{3n+1}$ (with excess m_{MACl}/m_{MAI} = 0.5) without the use of an antisolvent and (**E**) SEM images of the corresponding perovskite films

(*n*-value indicated in the top right). (**F**) *PCE*, (**G**) *FF*, (**H**) V_{oc} and (**I**) J_{sc} of analogously processed solar cells but with perovskite films grown using chlorobenzene as an antisolvent and (**J**) SEM images of the corresponding perovskite films (*n*-value indicated in the top right). Error bars represent the standard deviation as obtained from 8 devices. (**K**) Absorbance and (**L**) time-resolved photoluminescence for the perovskite films grown on PEDOT:PSS using different *n*-values with chlorobenzene as antisolvent. (**M**) Absorption profile as measured using photothermal deflection spectroscopy for $n = 7$ to determine the bandgap of 1.58 ± 0.01 eV.

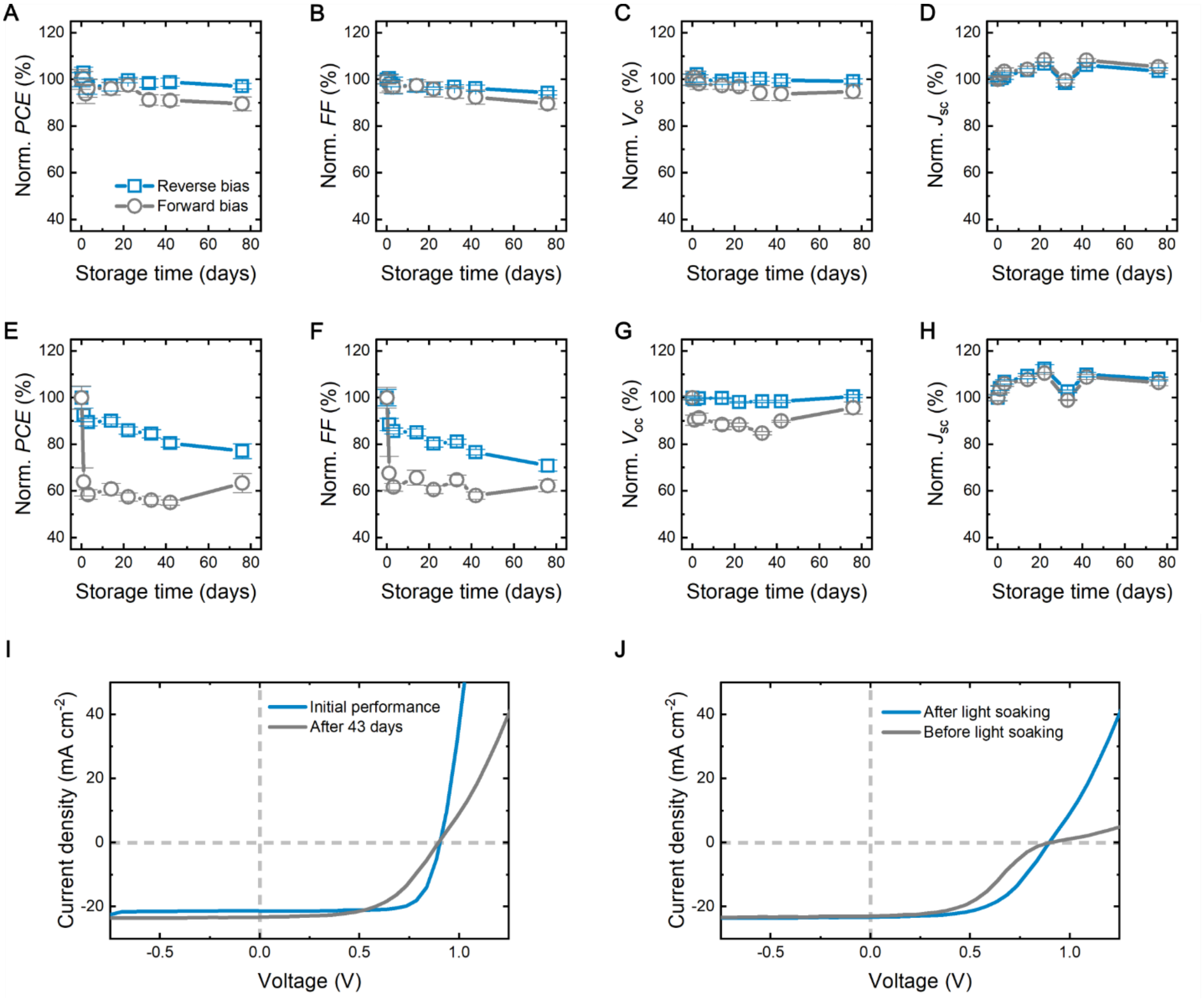

Figure S6: Storage stability. (**A**) Normalized *PCE*, (**B**) *FF*, (**C**) V_{oc} and (**D**) J_{sc} of solar cells (fabricated with $n = 7$ and $m_{MACl}/m_{MAI} = 0.5$) as a function of the time stored in the dark inside a nitrogen atmosphere. (**E**) Normalized *PCE*, (**F**) *FF*, (**G**) V_{oc} and (**H**) J_{sc} of unencapsulated solar cells (fabricated with $n = 7$ and $m_{MACl}/m_{MAI} = 0.5$) while stored in the dark under atmospheric conditions (on average 21 ± 2 °C and 60 ± 10 % RH). The PCEs were normalized to 14 %. Error bars represent standard deviations as obtained from 8 devices. (**I**) JV characteristics of the champion device on the first day and after degrading to 80 % of its initial performance after 43 days and (**J**) on day 43 before and after light-soaking for 15 minutes.

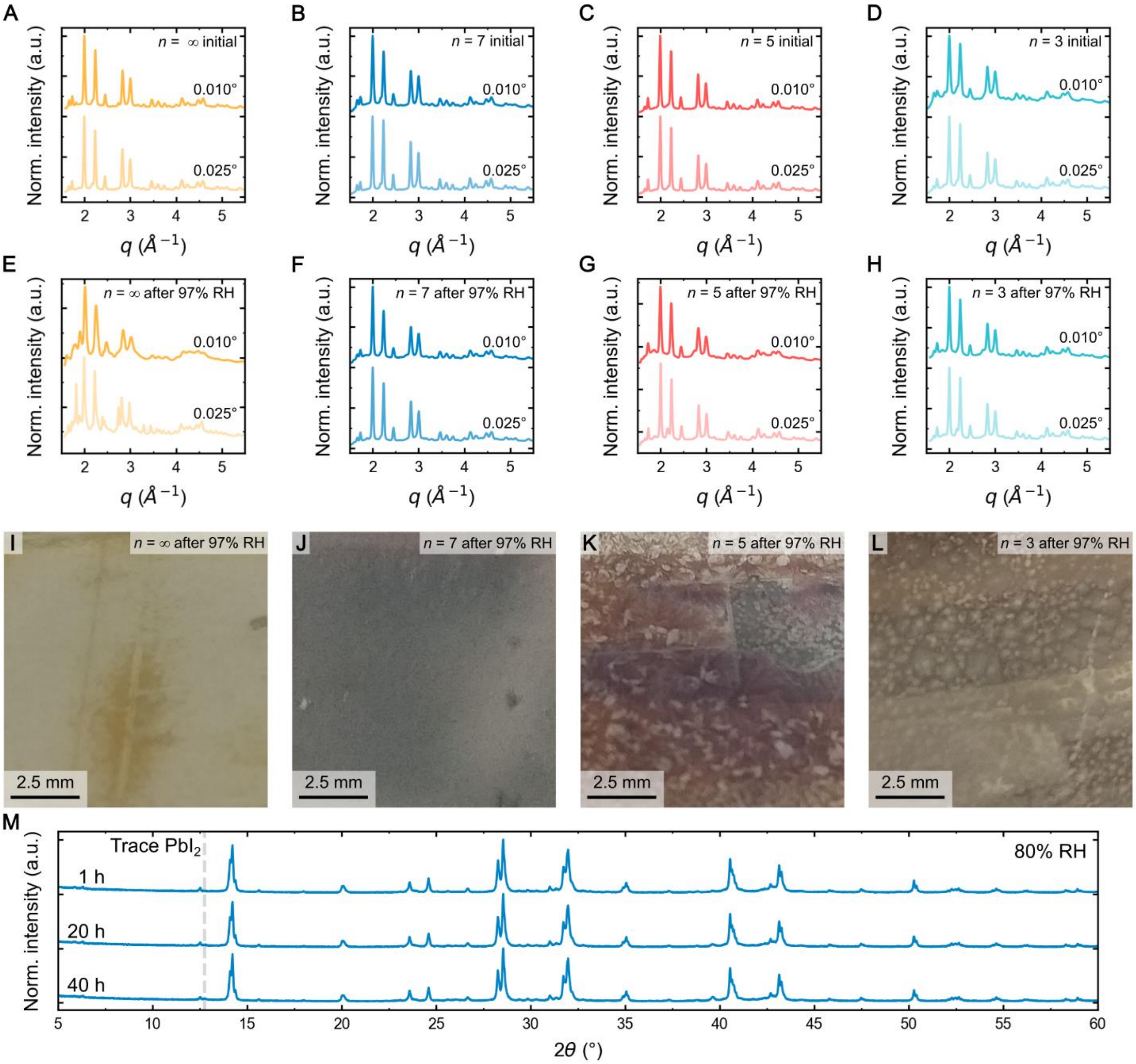

Figure S7: GIWAXS & XRD stability investigations. (**A**) Initial GIWAXS patterns of $n = \infty$, (**B**) $n = 7$, (**C**) $n = 5$ and (**D**) $n = 3$ films recorded at different angles of the incident X-ray beam (as indicated on the right) to probe different depths. Angles of 0.01 ° and 0.025 ° correspond to penetration depths of about 3 nm and > 230 nm, respectively (see calculations shown in Supplementary Note 2). Angles larger than 0.025 ° already started to probe the ITO layer underneath the perovskite. (**E-H**) Repetition of the same measurement after exposing the films to a high humidity of 97 % RH for about 10 h. All patterns were normalized to their maximum intensity. (**I-L**) Optical images of the degraded perovskite films after humidity exposure. (**M**) XRD pattern of optimized $n = 7$ perovskites when exposed to ambient air with 80 % RH for 40 h. Aside from the appearance of a minor trace of PbI_2, the pattern remains unchanged for the duration of the measurement. All patterns are normalized to the maximum intensity of the data recorded after 1 h. Both the GIWAXS and XRD measurements demonstrate how the $n = 7$ films are unaffected by these extreme conditions.

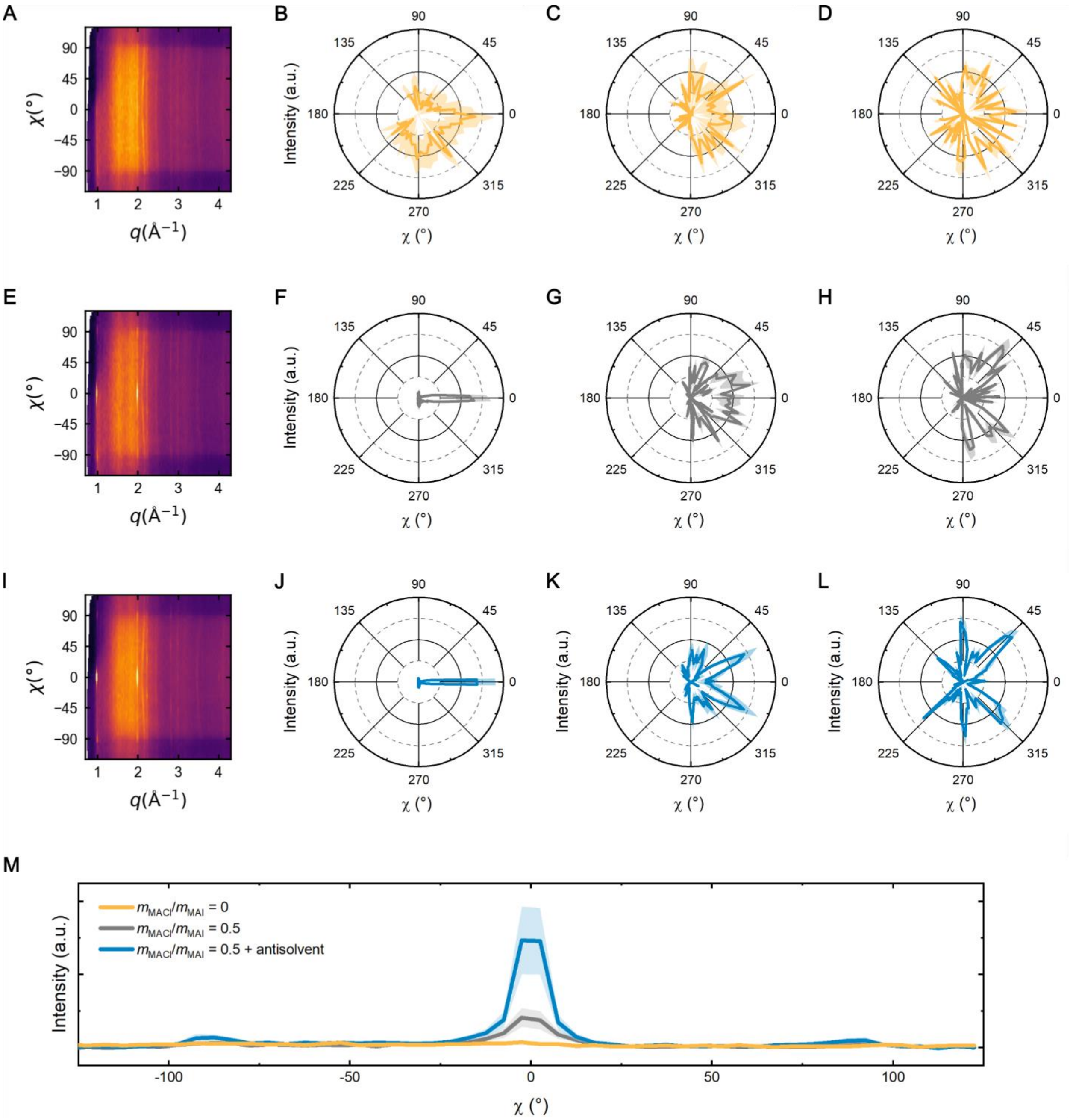

Figure S8: MACl-induced anisotropy. (**A**) Heatmap of the scattered X-ray intensity from perovskite films grown without MACl. Negative angles correspond to 180-360 °. (**B**) Anisotropy of the corresponding peaks at q = 1 Å^{-1} ((002) and (110)), (**C**) 2.2 Å^{-1} ((130), (114), and (222)), and (**D**) 2.8 Å^{-1} ((224) and (040)) is measured by regrouping the scattered intensity recorded on a 2D pixel detector as a function of q and azimuthal angle χ. (**E**) Same data for perovskite films fabricated with m_{MACl}/m_{MAI} = 0.5 and the (**F**) respective peak anisotropy at q = 1 Å^{-1}, (**G**) 2.2 Å^{-1} and (**H**) 2.8 Å^{-1}, as well as for (**I**) films made with m_{MACl}/m_{MAI} = 0.5 and toluene as an antisolvent with the (**J**) anisotropy of peaks at q = 1 Å^{-1}, (**K**) 2.2 Å^{-1} and (**L**) 2.8 Å^{-1}. The observed four- and six-fold symmetry at 2.2 Å^{-1} and 2.8 Å^{-1} occur since for each reflection (*hkl*), different lattice planes {*hkl*} can contribute, which are equivalent by symmetry. (**M**) Comparison of the peak anisotropy

at q = 1 Å^{-1} of the three analyzed samples. Shaded regions represent the standard deviation as obtained from averaging over the peak (Δq = 0.05 Å^{-1}) and subtracting the background.

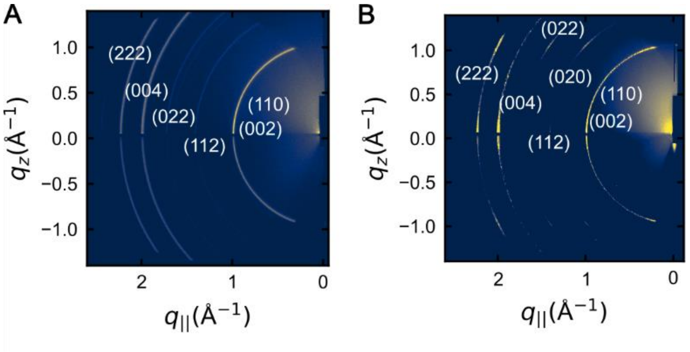

Figure S9: GISAXS. (**A**) GISAXS images of perovskite films as grown with m_{MACl}/m_{MAI} = 0 and (**B**) m_{MACl}/m_{MAI} = 0.5. Lattice planes corresponding to the diffraction peaks are indicated in parentheses. We note that while no diffraction signals can be observed below $q_{||}$ < 1 Å^{-1}, small quantities of superstructure phases may still be present below the detection limit.

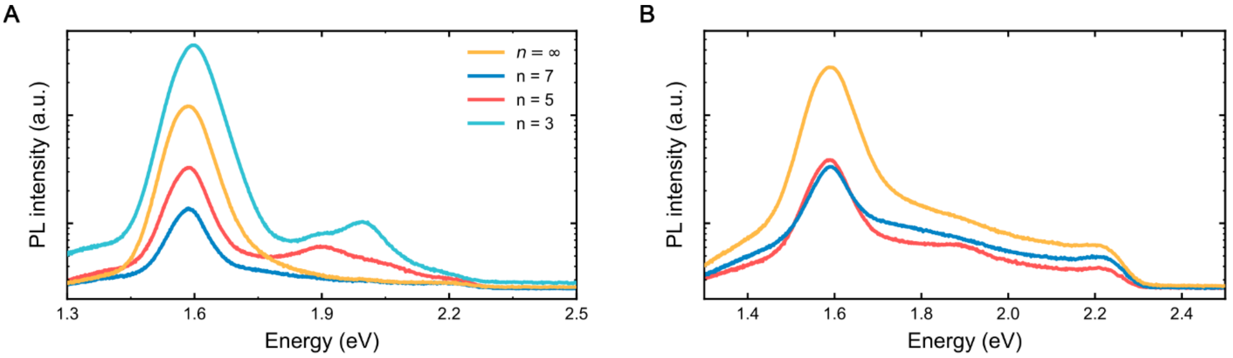

Figure S10: Photoluminescence. (**A**) Photoluminescence spectra for perovskite films of different n-value grown on PEDOT:PSS as recorded from the substrate side and the (**B**) perovskite-coated side. Samples with n = 3 and 5 exhibit additional higher-energy bands typical for lower-n 2D or quasi-2D phases (we note the log-scale for clarity). For measurements performed on the perovskite-coated side, an additional shoulder is observed. Since this shoulder is also present for $n = \infty$, we can attribute it to scattering from the perovskite surface, not to lower-n phases. The cutoff around 2.25 eV is due to the 550 nm longpass filter employed to avoid the signal from the excitation wavelength. We note that while the photoluminescence spectra of n = 7 does not display clear evidence of the existence quasi-2D phases, it is likely that a distribution of n phases is present below the detection limit.

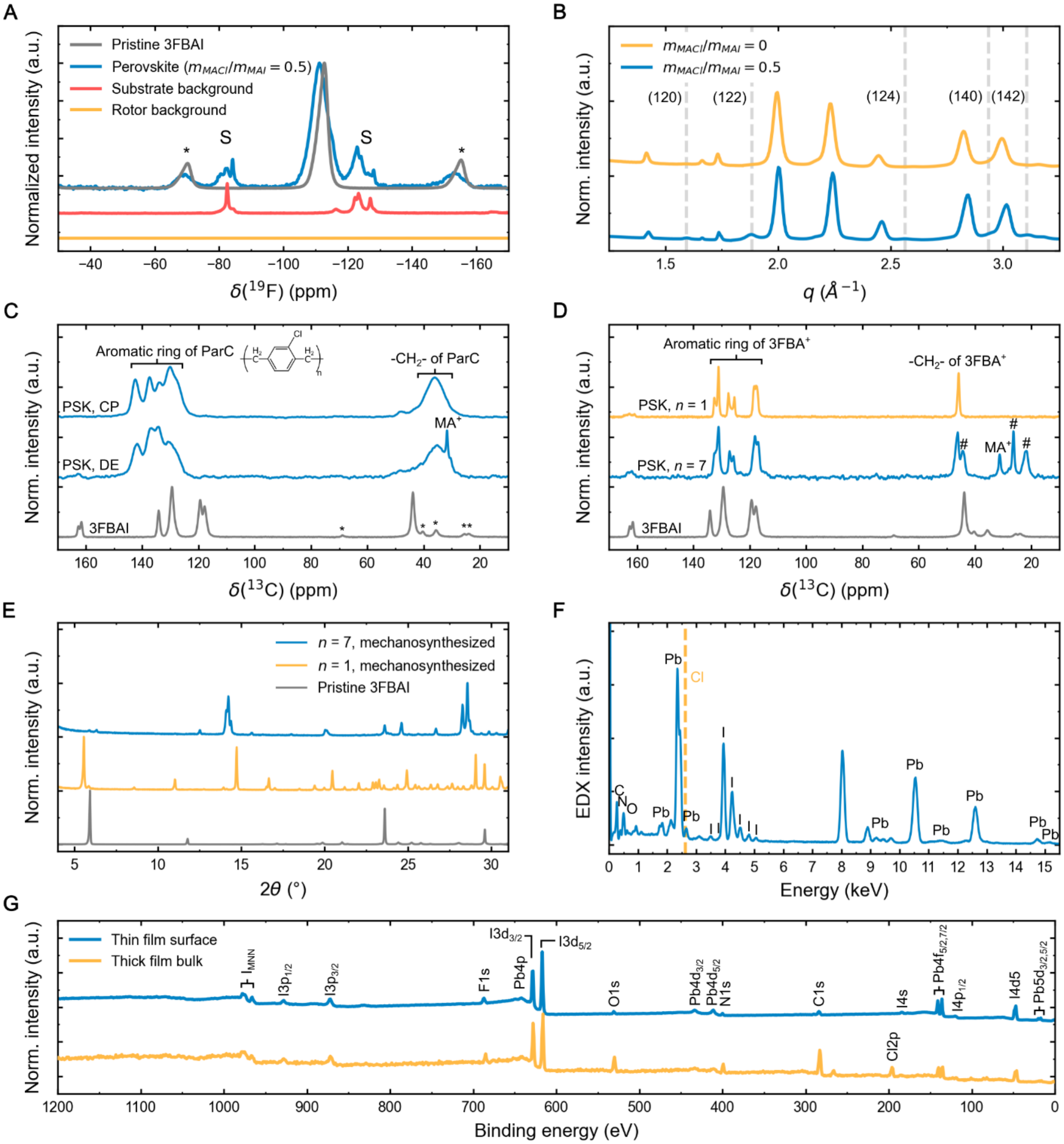

Figure S11: Chlorine investigation. (**A**) Comparison of the full ssNMR spectrum of the pristine 3FBAI salt, perovskite films fabricated on flexible parylene C substrates with m_{MACl}/m_{MAI} = 0.5. The observed shift in peak position shows that the 3FBAI has reacted to form a new local environment in the n = 7 perovskite film. 3FBA reacting is also evident from the substantial change of its longitudinal relaxation time (return to thermodynamic equilibrium after excitation) from T_1 = 424 s (for 3FBAI) to T_1 = 2.7 s (for films with m_{MACl}/m_{MAI} = 0.5) and T_1 = 2.0 s (for films with m_{MACl}/m_{MAI} = 0). Background spectra of the Parylene C substrate and the rotor are included to show that the peaks around a chemical shift of -82 ppm and -123 ppm (as indicated with an S) correspond to the fluorinated silane used during the fabrication of the substrate. Peaks at -68 ppm and -153 ppm (as indicated by asterisks) correspond to spinning sidebands which are copies of the main peak at -110 ppm. (**B**) Combined GIWAXS and GISAXS patterns of perovskite films

grown with and without MACl. The peaks observed for m_{MACl}/m_{MAI} = 0 can be indexed by the tetragonal space groups I4cm (#108) or I4/mcm (#140) typical for $MAPbI_3$, corresponding to the Glazer tilt system $a^0a^0c^-$. However, for films grown with m_{MACl}/m_{MAI} = 0.5, new peaks appear (as indicated by the grey dashed lines). These new peaks ((12*l*) and (14*l*) with *l* even) would be forbidden by these space groups and therefore indicate the coexistence of a P4/mbm space group (#127, $a^0a^0c^+$), likely due to the incorporation of trace amounts of Cl into the crystal structure. Corresponding lattice planes are indexed for the original tetragonal unit cell and indicated in parentheses. We note that while there is no evidence for mixed-*n* phases, the presence of trace amounts of low-dimensional structures, as well as the accumulation of 3FBA at grain boundaries or interfaces cannot be ruled out by the above XRD measurements. (**C**) ^{13}C MAS NMR spectra of thin films (m_{MACl}/m_{MAI} = 0) grown on flexible parylene C (ParC) substrates, as recorded using cross-polarization (CP) and direct excitation (DE) using an echo, as well as of (**D**) mechanosynthesized *n* = 1 and 7 PSK films. Peaks labelled with an asterisk and # indicate spinning sidebands or originate from trace polypropylene (grinding jar material), respectively. The spectrum of the pristine 3FBAI salt is given as a reference. (**E**) XRD pattern of the mechanosynthesized *n* = 1 and 7 PSK films, as well as the pristine 3FBAI salt. (**F**) General EDX spectrum of a selected area in the middle of the perovskite layer. The dashed line corresponds to the region where the K_α Cl peak would appear if it was visible. However, this region is overlapped by a much larger Pb peak, which corresponds to its parent peak at 2.34 keV (for the M_α emission line of Pb). (**G**) Full XPS survey spectrum of the optimized *n* = 7 perovskite films' surface and of a thick films bulk, both grown with m_{MACl}/m_{MAI} = 0.5. The Cl2p signal only appears inside the bulk.

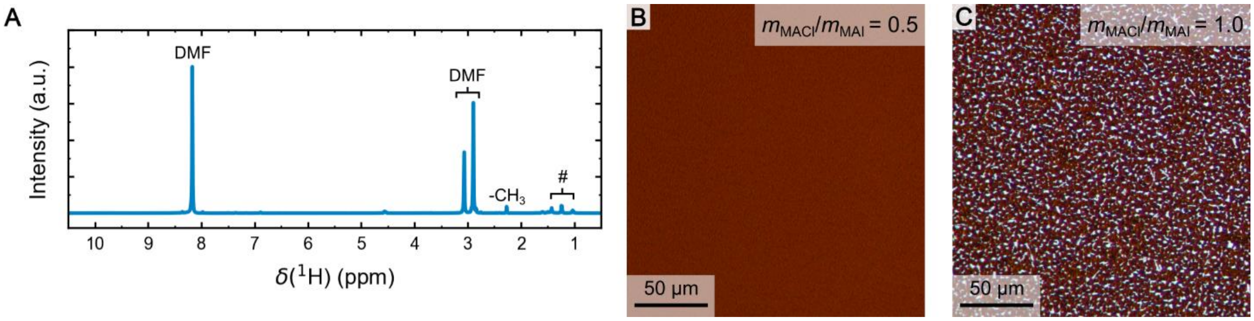

Figure S12: Gas evaporation. (**A**) Full liquid-state ^{1}H NMR spectrum of the condensed liquid generated during the evaporation of the precursor solution during annealing at 100 °C. Peaks labelled with # correspond to sample impurities. (**B**) Optical microscopy images of perovskite films with m_{MACl}/m_{MAI} = 0.5 and (**C**) m_{MACl}/m_{MAI} = 1.0. While the optimized ratio of 0.5 forms a compact pinhole-free film, too large of an excess of MACl results in poor film quality with numerous pinholes. This can be attributed to the excess MACl escaping through the bulk of the film via the gas phase, leading to hole formation.

Supplementary References

[1] D. Prochowicz, M. Franckevičius, A. M. Cieślak, S. M. Zakeeruddin, M. Grätzel, J. Lewiński, *J. Mater. Chem. A* **2015**, *3*, 20772.

[2] D. Prochowicz, M. Saski, P. Yadav, M. Grätzel, J. Lewiński, *Acc. Chem. Res.* **2019**, 3233.

[3] J. Gustafson, M. Shipilin, C. Zhang, A. Stierle, U. Hejral, U. Ruett, O. Gutowski, P. A. Carlsson, M. Skoglundh, E. Lundgren, *Science (80-.).* **2014**, *343*, 758.

[4] J. F. Young, *J. Appl. Chem.* **2007**, *17*, 241.

[5] J. Kieffer, V. Valls, N. Blanc, C. Hennig, *J. Synchrotron Radiat.* **2020**, *27*, 558.

[6] A. Z. Chen, M. Shiu, J. H. Ma, M. R. Alpert, D. Zhang, B. J. Foley, D. M. Smilgies, S. H. Lee, J. J. Choi, *Nat. Commun.* **2018**, *9*, 1.

[7] F. Mayr, **2020**, 1.

[8] J. Tauc, R. Grigorovici, A. Vancu, *Phys. Status Solidi* **1966**, *15*, 627.

[9] C. T. Chantler, *J. Phys. Chem. Ref. Data* **1995**, *24*, 71.

[10] R. Feidenhans'L, *Surf. Sci. Rep.* **1989**, *10*, 105.
